# Recent updates on nano-phyto-formulations based therapeutic intervention for cancer treatment

**DOI:** 10.32604/or.2023.042228

**Published:** 2023-11-15

**Authors:** ABHISHEK WAHI, MAMTA BISHNOI, NEHA RAINA, MEGHNA AMRITA SINGH, PIYUSH VERMA, PIYUSH KUMAR GUPTA, GINPREET KAUR, HARDEEP SINGH TULI, MADHU GUPTA

**Affiliations:** 1 Department of Pharmaceutics, School of Pharmaceutical Sciences, Delhi Pharmaceutical Sciences and Research University (DPSRU), Pushp Vihar, New Delhi, 110017, India; 2 Department of Pharmaceutical Sciences, Gurugram University, Haryana, 122003, India; 3 Department of Life Sciences, Sharda School of Basic Sciences and Research, Sharda University, Greater Noida, Uttar Pradesh, 201310, India; 4 Department of Biotechnology, Graphic Era (Deemed to be University), Dehradun, Uttarakhand, 248002, India; 5 Department of Pharmacology, Shobhaben Pratapbhai Patel School of Pharmacy & Technology Management, SVKM’s NMIMS, Vile Parle (West), Mumbai, 400056, India; 6 Department of Bio-Sciences and Technology, Maharishi Markandeshwar Engineering College, Maharishi Markandeshwar (Deemed to be University), Mullana, Ambala, 133207, India

**Keywords:** Phyto-formulations, Cancer, Nanocarriers, Nanotechnology, Clinical development

## Abstract

Cancer is a leading cause of death globally, with limited treatment options and several limitations. Chemotherapeutic agents often result in toxicity which long-term conventional treatment. Phytochemicals are natural constituents that are more effective in treating various diseases with less toxicity than the chemotherapeutic agents providing alternative therapeutic approaches to minimize the resistance. These phytoconstituents act in several ways and deliver optimum effectiveness against cancer. Nevertheless, the effectiveness of phyto-formulations in the management of cancers may be constrained due to challenges related to inadequate solubility, bioavailability, and stability. Nanotechnology presents a promising avenue for transforming current cancer treatment methods through the incorporation of phytochemicals into nanosystems, which possess a range of advantageous characteristics such as biocompatibility, targeted and sustained release capabilities, and enhanced protective effects. This holds significant potential for future advancements in cancer management. Herein, this review aims to provide intensive literature on diverse nanocarriers, highlighting their applications as cargos for phytocompounds in cancer. Moreover, it offers an overview of the current advancements in the respective field, emphasizing the characteristics that contribute to favourable outcomes in both *in vitro* and *in vivo* settings. Lastly, clinical development and regulatory concerns are also discussed to check on the transformation of the concept as a promising strategy for combination therapy of phytochemicals and chemotherapeutics that could lead to cancer management in the future.

## Introduction

**FIGURE 1:**
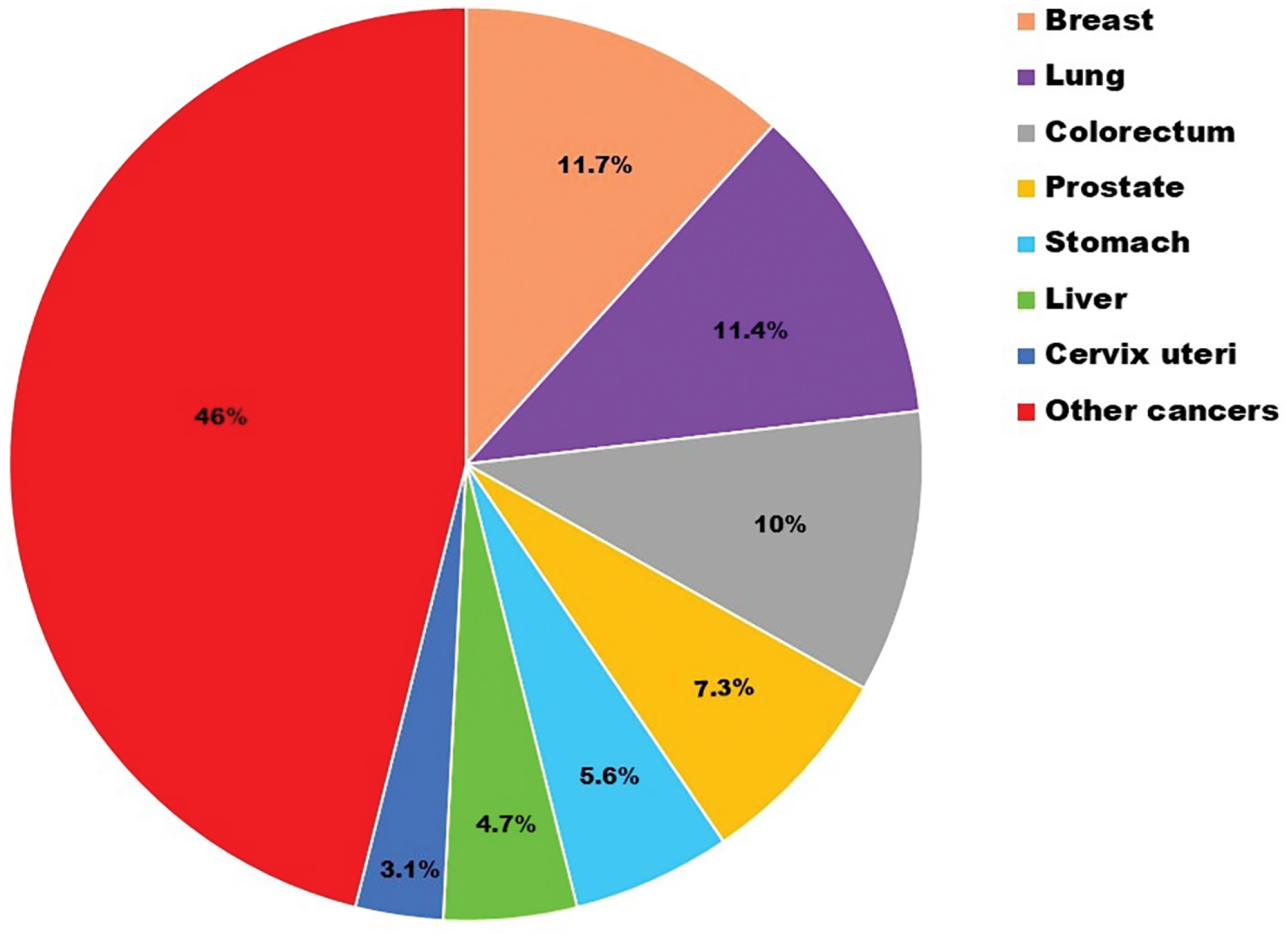
Estimated number of new cases in 2020 in the world for both sexes [9].

Cancer was and still is one of the dreadful diseases from ancient times. Though ages have witnessed many advances in cancer therapy, the rising cases indicate the call for much deep understanding and newer therapeutic moieties or remolding of the existing therapy to control the prevalence of the disease. International Agency for Research on Cancer (IARC) has conducted a survey in 185 countries on all age groups, sex categories, and 36 types of cancers to make available a global cancer burden database based on cancer incidents and cancer mortalities [1]. This data is alarming enough (Fig. 1) to look for new treatments that can outshine conventional treatments. Conventional therapy includes surgery, chemotherapeutic anticancer drug delivery, or radiation therapy. Cancer cells exhibit a high rate of proliferation, leading to their expansion and concurrent infiltration of adjacent cells. Conventional therapy is associated with affecting/destroying healthy cells during treatment, which can also lead to lethal effects [2].

Nanotechnology is quickly acquiring worldwide consideration as a crucial part of biomedical research explicitly aimed at cancer theranostics. The unique properties of nano-formulations include a tailorable surface and a high surface area-to-volume ratio. This system facilitates efficient adsorbing and encapsulating of the therapeutic agent, including phytochemicals, for site-specific drug delivery or passive delivery. Additionally, these nanoformulations condense the possibility of systemic toxicity and enhance the bioavailability with programmed release to the target site. Some common examples of such carrier systems are liposomes, polymeric nanoparticles (NPs), and polymeric micelles [3]. Nowadays, healthcare professionals are looking up to the traditional medicinal system to redesign the current therapeutics by combining the knowledge of the herbal with technology. Herbal medicine, or phytomedicine, uses bioactives derived from plants/herbs to promote wellbeing [4–6]. Severe synthetic medicines-related adverse effects and poor therapeutic choices for acute diseases make plant-based medications popular worldwide. Many clinical trials are underway to check out the chemotherapeutic potential of plant-based chemicals [7,8]. Natural plant resource phytochemicals can be used alone or in combination with anticancer drugs for better cancer theranostic over the conventional approach.

Numerous phytochemicals have shown prominent anticancer activity in preclinical models, including Curcumin, irinotecan, marijuana, and epipodophyllotoxin. Secondary metabolites like terpenes, alkaloids, terpenoids, flavonols, phenols, etc., are a vital source of anticancer compounds undergoing clinical trials [10]. The secondary metabolites help in the regulation of natural processes within the body. These mechanisms encompass the inhibition of overexpressed proteins, enzymes, amino acids, and hormones. Phytochemicals additionally expedite the synthesis of defensive enzymes. Also, they regulate different pathways, proving their role in relative oxygen generation [11]. Clinical results are still in the research phase, and it is not easy to find an optimal combination treatment [12].

Despite the encouraging potential of phytochemicals as an anticancer agent, several concerns still need to be pondered. The primary issues associated with phytochemicals are their low solubility, susceptibility to first-pass metabolism, narrow therapeutic window, and lack of specificity. Low solubility hampers the characteristic of permeability and ultimately affects bioavailability. Phyto-formulations given via the oral route may go through the first-pass effect and can get degraded before their therapeutic action. Apart from these pharmacological concerns, another concern associated with phytochemicals is being natural components they may be recognized as supplements and may be taken up by normal healthy tissue [13]. In addition, another hurdle in the effective use of phytochemicals is the chance of the development of resistance via multiple pathways. Indeed, nanotechnology can help to overcome the pitfalls of conventional phytochemicals-based therapy. Still, detailed research, including a clinical trial, is the call of time to make the phytochemical-based nano-therapy a success [14,15]. The present review focuses on nanotechnology-based phyto-formulations and their application in cancer treatment. Also, a brief of clinical trial status and future perspective of phytochemical-based nanotherapeutics is discussed.

## Phytochemicals for Cancer Therapy

Progressive cell division and the ability of the cells to spread to other bodily areas are the primary characteristics of cancer. Various factors are mainly responsible for the cause of cancer, including epigenetic modifications in the genes, environmental factors like UV radiation, smoking, exposure to harmful chemicals, etc. Epidemiological studies revealed that incidences of cancers are increasing worldwide along with an increase in the problem of drug resistance. This emphasizes researchers’ interest in developing strategies related to cancer treatment [16,17]. Phytochemical-based foods or treatments have taken a massive lead over the past few decades. Researchers are keen to approach a unique treatment method for diseases like neurodegeneration, obesity, and cancer. Plant-based food, including vegetables, fruits, and whole grains, contribute to positive health outcomes because they are rich in diverse phytochemicals of great importance to human health [18]. Today, research in the field of cancer is mainly transferred from synthetic molecules to phytoconstituents (Table 1) because of their easy availability, economical, less side/toxic effects, and combination therapy. One of the main drawbacks associated with anticancer chemotherapy regimens is the emergence of resistance that occurs over the course of ongoing treatment. Phytoconstituents play the most significant role in combating chemo-resistance by modulating targets like various signaling events and many regulators involved in drug transport, cell survival, epithelial-mesenchymal transition, apoptosis, etc. The phenomenon of chemo-resistance is observed when there is an excessive expression of efflux pumps, namely MDR1, p-gp, LRP, and BCRP. Phenolic phytochemicals, including genistein, EGCG, quercetin, emodin, and resveratrol, have been found to enhance the cytotoxic potential of various cytotoxic drugs such as paclitaxel, docetaxel, gemcitabine, 5-FU, vinblastine, vincristine, cisplatin, doxorubicin, TRAIL, temozolomide, and sorafenib. This enhancement is achieved through the modulation of multiple signalling pathways and the targeting of molecules that confer resistance to these drugs [19]. However, recent research has been primarily dedicated to investigating the utilisation of formulations containing phytochemicals in combination with conventional anticancer medications. The aim is to restore the sensitivity to chemotherapy in cases where resistance has developed due to prolonged use of these chemotherapeutic agents. Therefore, these combination therapies improve healing efficacy and result in favorable treatment outcomes [20]. Thus, this section mainly discusses the role of essential phytochemicals in cancer treatment.

**Table 1:** Comprehensive detail of phytochemical-based formulations used for targeting and treatment of cancer

S. No.	Name of plant species (Family)	Plant part used	Extract type	Formulation type	Cell line/Animal model	Inference	Reference
1.	*Adiantum capillus veneris *L. (Pteridaceae)	Leaves	Methanol	AuNPs	MCF7 and BT47 (Human breast cancer cell lines)	Antiproliferative activity (+) Apoptosis	[78]
2.	*Allium sativum *L. (Amaryllidaceae)	Cloves	Aqueous	AgNPs	MCF-7	(−) Cell viability at a dose of 100 µg/mL	[79]
3.	*Alternanthera sessilis (*L*.) R.Br. ex-DC*. (Amaranthaceae)	Leaf	Aqueous	AuNPs	HeLa (Cervical cancer cell line)	(+) Cytotoxicity and apoptosis by modulating intrinsic apoptotic pathway	[80]
4.	*Artemisia tournefortiana Rchb*. (Asteraceae)	Aerial parts	Ethanol	AgNPs	HT29 (Human colon adenocarcinoma cancer cell line)	↑ Apoptosis in a dose-dependent manner	[81]
5.	*Atropa acuminata Royle ex Lindl*. (Solanaceae)	Seeds	Aqueous	AgNPs	HeLa	Strong inhibitory activity against cancer cells. Production of ROS and ↓ levels of GSH	[82]
6.	*Bauhinia purpurea *L. (Fabaceae)	Leaf	Aqueous	AuNPs and AgNPs	(A549) Lung carcinoma cell line	Possess momentous anticancer effect	[83]
7.	*Borago officinalis *L. (Boraginaceae)	Leaves	Aqueous	AgNPs	A549 and HeLa	Possess cytotoxic activity	[84]
8.	*Camellia sinensis (*L*.) Kuntze* (Theaceae)	Leaf	Aqueous	AgNPs	HT-29, MCF-7, and MOLT-4 (Human leukemia cancer cell line)	Possess antiproliferative activity	[85]
9.	*Catharanthus roseus (*L*.) G. Don* (Apocynaceae)	Leaves	Aqueous	AgNPs	HepG2 (Liver hepatocellular carcinoma cell line)	(−) Proliferation of cancerous cells (+) Apoptosis due to MMP loss, arrest in the cell cycle and DNA damage	[86]
10.	*Clausena lansium (Lour.) Skeels* (Rutaceae)	Fruit peels	Aqueous	ZnO NPs	SH-SY5Y (Neuroblastoma cancer cell line)	Regulates autophagy (Beclin-1, LC3-I, -II, and ATG4B) and apoptotic proteins (Bax, Bcl-2 and Caspase-3) Production of ROS in cells ↓ Cell viability and stability	[87]
11.	*Curcuma wenyujin Y. H. Chen & C. Ling* (Zingiberaceae)	Rhizome	Aqueous	AuNPs	A498 (Human renal cell carcinoma cell lines)	(+) Apoptosis by ↑ the expression of apoptotic caspase-3, 9, Bid and Bad and ↓ levels of anti-apoptotic proteins like Bcl-2 and Bcl-XL	[88]
12.	*Cynara scolymus* L.	Leaf	Aqueous	AgNPs	MCF7	Broad-spectrum anticancer activity, thereby causing damage to mitochondria and ROS generation, which shows significant ↓ in cell migration, expression of Bax and suppression of Bcl-2	[89]
13.	*Deverra tortuosa (Desf.) DC*. (Apiaceae)	Aerial parts	Aqueous	ZnO NPs	Caco-2 (Human colorectal epithelial adenocarcinoma) and A549	Cytotoxic to cancer cells	[90]
14.	*Erythrina suberosa (Roxb.)* (Fabaceae)	Leaves	Aqueous	AgNPs	A-431 (Osteosarcoma cell line)	Possess excellent anticancer activity at an IC_50_ value of 74.02 ± 2.4 µg/mL	[91]
15.	*Ficus krishnae C. DC*. (Moraceae)	Stem bark	Aqueous	AgNPs	SKOV3 (Ovarian cancer cell lines)	Possess cytotoxic activity at an IC_50_ value of 22.85 μg/μL	[92]
16.	*Hibiscus rosa-sinensis* (Malvaceae)	Leaves	Aqueous	AgNPs	SNU-387 (Pleomorphic hepatocellular carcinoma), LMH/2A (Hepatic ductal carcinoma), McA-RH7777 (Morris hepatoma), and N1-S1 Fudr (Novikoff hepatoma)	↓ Cell viability and ↑ Anti-liver cancer activities in a dose-dependent manner against cancerous cell lines without any cytotoxicity to the normal cells (HUVEC) The IC_50_ of AgNPs were 477, 548, and 605 mg/mL	[93]
17.	*Lantana camara *L. (Verbenaceae)	Root	Aqueous	AuNPs	MBA-MB-231 (Human breast cancer cell line) and Vero (Normal kidney cells)	Possess cytotoxic effects and exhibit apoptosis in a dose-dependent manner and membrane leakage (+) Apoptosis and fragmentation of DNA	[94]
18.	*Litchi chinensis Sonn*. (Sapindaceae)	Leaves	Aqueous	AgNPs	HEp-2 and MCF-7	Possess anticancer activity by causing cell death	[95]
19.	*Mangifera indica *L**. (Anacardiaceae)	Seed	Aqueous	AuNPs	Human gastric cancer cell line	Possess anticancer and antiangiogenic (by ↓ Ang-1/Tie2 pathway) activity ↓ Cancer cell growth	[96]
20.	*Marsdenia tenacissima (Roxb.) Moon*	Leaves	Aqueous	AuNPs	HepG2 cells	Possess cytotoxic activity (+) apoptosis by enhanced ROS production alters MMP and (−) the migration ↑ Expression of Bax, caspase-3 and 9 ↓ Expression of Bcl-2 and Bcl-XL	[97]
21.	*Origanum glandulosum Desf*. (Lamiaceae)	Aerial parts	Essential oil	Nanocapsules	HepG2 and THLE2 (Human liver cancer cell lines)	Strongest cytotoxic activity in comparison to any essential oil	[98]
22.	*Phoenix dactylifera *L. (Arecaceae)	Root hair	Ethanol	AgNPs	MCF7	↓ Cell viability (+) apoptosis Arrest progression of the cell cycle in sub-G1 and S-phase and unable the cells to enter the M phase	[99]
23.	*Brassica napus *L. (Brassicaceae)	Flower pollen	Aqueous	AgNPs	MDA-MB-231 and MCF7	↓ Cancer cells viability ↑ apoptotic cells % Strong antioxidant effects and potentially suppress carcinogenesis via ↓ expression levels of VEGF	[100]
24.	*Rhamnus triquetra (Wall.) Brandis* (Rhamnaceae)	Leaves	Aqueous	NiO NPs	HepG2 and HuH7 (Hepatic cancer cell lines)	Possess potential anticancer activity	[101]
25.	*Rubia cordifolia *L. (Rubiaceae)	Leaf	Aqueous	ZnO and CeO_2_ NPs	MG-63 (Human osteosarcoma cell lines)	It encourages loss of cell membrane integrity, cell death, and apoptosis	[102]
26.	*Salvia miltiorrhiza Bunge* (Lamiaceae)	Leaf	Aqueous	AgNPs	LNCaP (Human prostate cancer cell lines)	(+) Cytotoxicity, ROS and apoptosis by modulating the expression of intrinsic apoptotic proteins like Bcl-2, Bax, Bcl-xl and caspase-3	[103]
27.	*Siberian ginseng (Eleutherococcus senticosus (Rupr. *&* Maxim.)* (Araliaceae)	Leaves and stems	Aqueous	AuNPs	B16 (Murine melanoma cell lines)	Possess anticancer activity and produce ROS and ↓ MMP ↑ Bid, Bad, caspase-3 and 9 ↓ Bcl-2 gene expression	[104]
28.	*Spinacia oleracea *L. (Amaranthaceae)	Leaf	Aqueous	AuNPs	Ishikawa, KLE, HEC-1-A, and HEC-1-B cell lines	IC_50_ was found to be 341, 335, 316, and 325 mg/mL. The best finding of anti-endometrial cancer potentials was determined in the HEC-1-A cell lines	[105]
29.	*Vetex negundo *L. (Lamiaceae)	Leaves	Ethanol	AuNPs	AGS (Human gastric adenocarcinoma hyperdiploid cell line)	↓ Proliferation of cancerous cells (+) Apoptosis by activating caspase-3 and Bax	[106]
30.	*Zataria multiflora Boiss*. (Lamiacea)	Leaves	Aqueous	AgNPs	HeLa	↓ Cell viability with chromatin fragmentation (+) Activates caspase-3/9 apoptotic pathway (−) Cell metastasis by ↓ levels of MMP and VEGFA expression	[107]

### Polyphenols

One of the largest groups of phytochemicals in the plant kingdom is polyphenols. They are bioactive compounds containing multiple phenolic groups in their structure, mainly classified as natural, synthetic, or semisynthetic compounds [21]. Polyphenols are a huge source of antioxidants, and this feature contributes to their mechanism of action as they scavenge free radicals or reactive oxygen species (ROS) and lower their levels in the human body [22,23]. Several studies have reported that various plant-derived natural polyphenols can protect normal cells directly or indirectly from the induction of neoplastic transformation due to xenobiotics and carcinogenic factors, thereby decreasing the risk of developing cancer [24]. Previous reports have established that in prostate cancer, polyphenolic compounds act as a chemopreventive agent due to their antioxidant or pro-oxidation activity, which is mainly contributed by reducing ROS generation, increasing enzyme activity specifically for antioxidation, and lastly by inducing cytotoxic effects.

Moreover, polyphenolic compounds also modulate androgen receptors by inhibiting their function or expression. In addition, polyphenolic compounds activate various signaling pathways in prostate cancer, like PI3K, Akt, ERK1/2, FOXO, GSK-3β, RTK, etc., ultimately suppressing cancer [25,26]. Polyphenols induce cell cycle arrest by downregulating various proteins, including cdc25, CDK2 and CDK4, cyclin E, and cyclin D1, and upregulating the proteins that code for tumor suppressor genes such as p21, p53, etc. They also induce apoptosis by activating caspase -3, -9, cyt-c, or Bax proteins [27].

Resveratrol, chemically known as 3, 5, 4′-trihydroxy-trans-stilbene, is a natural polyphenol in the stilbene family. Familiar plant sources of resveratrol include peanuts, almonds, berries-raspberries, mulberries, blueberries, grapes, beans, etc. [28]. Researchers have reported that resveratrol inhibits skin cancer development and can delay its onset. It protects DNA from ROS [29]. For example, resveratrol protects DNA from free radical generation and scavenges ROS, superoxides, and hydroxyls produced in cancerous cells—a cascade of events related to tumor initiation [30]. According to Pereria and colleagues, resveratrol lessens pancreatic acinar cell necrosis and early intracellular trypsin activation. In addition to this, resveratrol also inhibits NF-kB signaling leading to anti-inflammatory effects after oral administration [31]. Zhang and colleagues stated that the co-encapsulated delivery of resveratrol and docetaxel nanoliposome combination exhibited higher cytotoxicity and increased the activity caspase-3, leading to cell death in prostate cancer. Moreover, this nanoliposome combination also decreases the growth of tumors in PC3-bearing Balb/c nude mice analyzed by a change in cell proliferation and apoptosis parameters [32]. Therefore, having such a combination of conventional drugs along with phytochemicals could serve great potential in synergistically fighting cancer.

**FIGURE 2:**
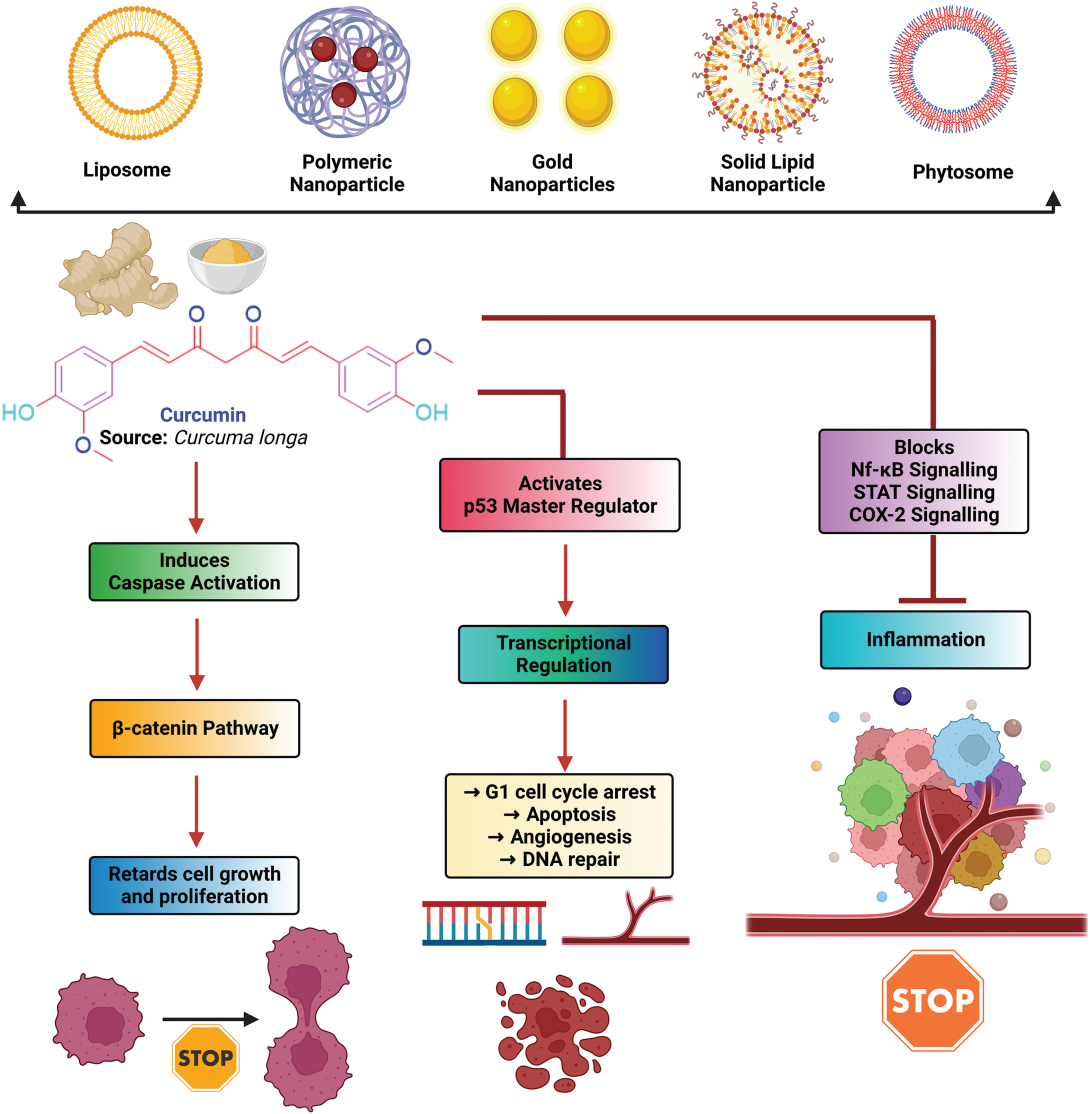
Molecular mechanism of anti-cancer action of curcumin [46].

Curcumin, obtained from *Curcuma longa* (known as turmeric), is a yellow-colored pigment mainly used as a traditional spice and food coloring agent [33,34]. Curcumin has a unique role in protecting biomembranes against peroxidative damage [35]. Several studies have reported that Curcumin (12 g/day for three months) activates apoptotic pathways against various cancer cell lines, including kidney, colorectal, prostate, pancreatic, and breast, showing antiproliferative activity [33]. Curcumin at a dose of 0.1–3 mg/kg body weight was shown to decrease the enzyme, telomerase reverse transcriptase, and decreased Bcl-2 expression. According to earlier studies, Curcumin’s low stability and insolubility in water have been blamed for reducing its bioavailability. Therefore, nanoencapsulation of Curcumin emerged as an effective strategy to enhance its therapeutic efficacy and bioavailability in combination with conventional chemotherapeutic agents [36]. With some selected phytochemical combinations (resveratrol, quercetin, epigallocatechin-3-gallate, and piperine), Curcumin exerts synergistic effects in cancer treatment to improve its clinical efficacy [37]. Fig. 2 represents a molecular mechanism of anticancer action of Curcumin.

Naringenin is an essential anticancer polyphenol. It mainly occurs in citrus fruits like lemons, oranges, tomatoes, etc. Naringenin generally induces cytotoxicity and apoptotic effects, thereby preventing the proliferation of different cancerous cells. Naringenin also helps downregulate the levels of pro-inflammatory mediators such as ICAM-1, COX-2, TNF-α, etc. Despite being a valuable phytochemical to target cancer, its *in vivo* bioavailability is very low due to its hydrophobic nature. Therefore, to overcome this implication and to enhance its bioavailability, the phytochemical in a delivery system at a nanoscale range was formulated [38]. Akhter et al. reported that naringenin-loaded PLGA polymeric NPs increased the cytotoxicity in pancreatic cell lines compared to only naringenin [39]. Another study also reported the beneficial effects of the phytochemical nano-based formulation; Yildirim et al. have established that intelligent polymeric NPs carrying naringenin (SPNPs) improve bioavailability and increase therapeutic effects in breast cancer. Moreover, SPNPs positively affect cell cycle arrest and apoptosis induction in breast cancer cells [40].

Quercetin belongs to the family of flavonoids. It is an excellent antioxidant by affecting ROS, glutathione, enzyme activity, and regulation of various signal transduction pathways, including AMPK, MAPK, and NRFB. Quercetin exhibits anticancer potential by inducing (a) cell cycle arrest G2/M phase in various cancer cell lines like U937, 232B4, SKOV3, etc.; (b) intrinsic apoptotic pathway; (c) autophagy and (d) inhibitory effects on angiogenesis [41]. Lakshmi et al. have reported the anticancer therapeutic implication of quercetin-mediated gold nanoclusters in lung cancer cells and established that nanoclusters have more significant and better cytotoxicity toward cancerous cells. Moreover, it also abolishes ROS generation stating antioxidant activity [42]. The Elsayed et al. [43] have documented the positive effects observed *in vivo* of quercetin encapsulated chitosan functionalized copper oxide nanoparticles (NPs) in breast cancer. These effects were attributed to the induction of apoptosis, mediated by an upregulation of the p53 gene, ultimately resulting in the death of mammary cancer cells. In addition, it concurrently downregulates the PCNA gene, resulting in a decrease in the rate of proliferation of cancer cells.

Kaempferol is an aglycone flavonoid yellow-colored compound abundantly found in various plant parts, including fruits, leaves, seeds, etc. It exhibits anticancer potential by triggering cell death, arrests in the cell cycle at phase G2/M, and downregulating the PI3K/AKT signaling pathway. Moreover, it also possesses antioxidant activity by reducing ROS metabolism, disrupting molecular mechanisms of proteins associated with cancer-related expression, including Nrf2 and Keap1 [44]. Several studies have reported the beneficial roles of kaempferol in targeting cancer. Govindaraju et al. investigated and reported that gold nanoclusters of kaempferol are cytotoxic to lung cancer cells characterized by nuclear damage. Moreover, kaempferol gold nanoclusters also inhibited the proliferation and migration of cancerous lung cells [45].

Aghazadeh et al. [47] reported that kaempferol-based nanostructured lipid carriers (KNLC) moderately inhibit the proliferation of breast cancer cells. In addition, combining KNLC with paclitaxel shows a synergistic effect leading to the strengthening of apoptosis and arrest in the progression of the cancer cell cycle at the sub-G1 phase.

Myricetin is a flavonoid mainly found in teas, berries, wines, etc. The anticancer activity of myricetin is mainly due to a reduction in MMP-2/9 activity. Reduction in mTOR activation induces apoptosis and downregulates MDR-1 [48]. Khorsandi et al. found that myricetin-loaded solid lipid NPs diminish colony formation in colorectal cancer cells. Moreover, it also elevates the expression levels of Bax and AIF while decreasing the levels of Bcl-2. It also helps in decreasing MMP [49].

Rutin is a unique antioxidant flavonoid in cereals, vegetables, and fruits. It serves as a promising natural phytochemical in the prevention of cancer by following molecular mechanisms; (a) modulation of cancer signaling pathways like MAPK, PI3K/Akt, JNK, etc., (b) inducing apoptosis by stimulating NF-kB pathway and cell cycle arrest in G2 and G1 phase by modulating GSTP1 and Cyp1A1 polymorphism, (c) reducing MMP [50]. Saleemi et al. have shown that zinc oxide NPs with bioflavonoid rutin possess anticancer effects against breast cancer cells by having higher antiproliferative activity than pure rutin. The primary molecular mechanism behind cytotoxicity is the release of intracellular zinc ions and ROS species production [51]. Additionally, head and neck cancer cells were said to resist the anticancer actions of rutin nanocrystals. In addition to this, it also decreases mRNA expression of Bcl-2 and activates a mitochondrial-dependent apoptotic pathway in cancerous cells [52].

Epicatechin is a flavonoid prominently found in black and green tea consumed globally. It possesses anti-tumour and chemopreventive properties due to its oligomeric structure. Epicatechin modulates various cancer pathways, including inhibition of the HGF/Met signaling pathway in breast cancer cells. Moreover, it also suppresses MMPs and other downregulating extracellular scaffolds, resulting in antiangiogenic activity [53]. Perez-Ruiz et al. have demonstrated the beneficial effect of encapsulating epicatechin with lecithin-chitosan NPs against breast cancer cells by inhibiting cell proliferation and encapsulating epicatechin as NPs increase the cytotoxic activity of epicatechin itself [54].

**FIGURE 3:**
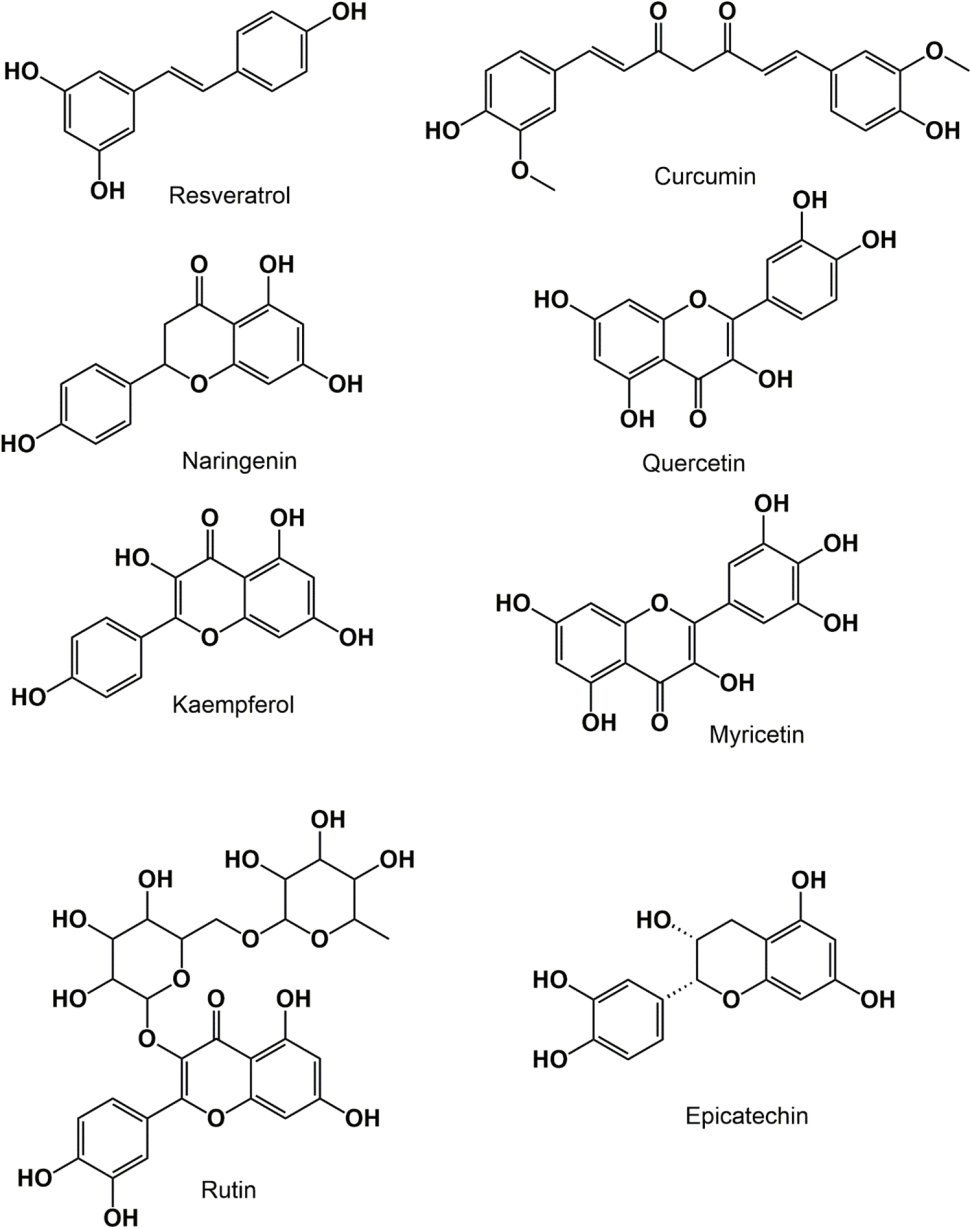
Chemical structures of various anticancer polyphenols.

Phenolic compounds have shown significant effects as cytotoxic phytochemicals against cancer, including induction of apoptosis and antiproliferative activity, and target various aspects of cancer (metastasis, proliferation, differentiation, and angiogenesis). Benzoic acid and phenolic acid are the major phenolics well-versed in different *in vitro* and *in vivo* models [55,56]. The chemical structures of various anticancer phyto-constituents are represented in Fig. 3.

### Alkaloids

**Figure 4:**
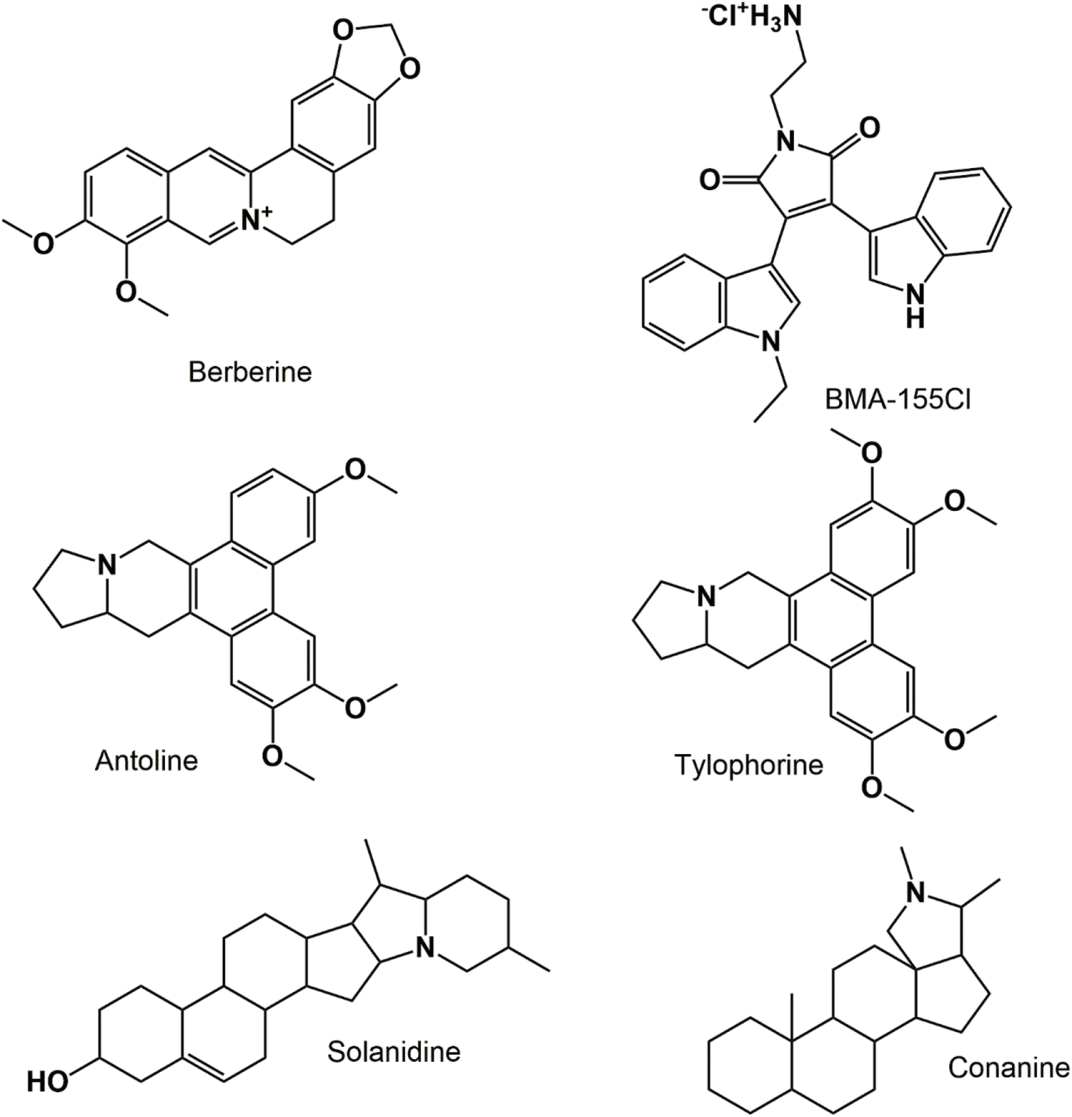
Chemical structures of various anticancer alkaloids

Alkaloids are naturally occurring essential compounds mainly of plant-based origin and are found particularly in specific families of flower-bearing plants. Alkaloids contain a cyclic structure with at least one essential nitrogen atom [57]. Various alkaloids (Fig. 4) are well-versed and studied worldwide as potential chemotherapeutic agents, including vinblastine, vincristine, camptothecin, piperine, berberine, etc. Vinblastine (Indole alkaloid) exhibits cytotoxic activity against several cancer cell lines by activating a cascade of proteins in the apoptotic signaling pathway, thereby inducing apoptosis, necroptosis, and autophagy [58]. Vinblastine is characterized as an autophagy maturation inhibitor; it enhances apoptosis in the human colon (LS174T) and human hepatocarcinoma (HepG2) cell lines synergistically in combination with nano liposomal C6-ceramide leading to the accumulation of autophagic vacuole and reducing autophagy maturation of the cells. Another indole alkaloid, Arctigenin, targets the senescence of cells in gall-bladder cancer by remarkably reducing the expression of EGFR levels through rapidly accelerated fibrosarcoma (RAF)-mitogen-activated protein kinase (MEK)-extracellular-regulated kinase (ERK) signaling pathway. It also induces cell death and arrests cell-cycle progression at G1/G0 phase by downregulating the levels of EGFR [59].

Various isoquinoline alkaloids possess antiproliferative activity by autophagy-influenced apoptosis mediated through the ATG5 gene. Berberine, an isoquinoline alkaloid, reduces colon tumor development by inhibiting the cyclooxygenase-2 (COX-2) enzyme. It exhibits anti-tumor activity against EAC cells by inhibiting the biosynthesis of RNA, DNA, and protein *in vitro* [60]. According to Wang et al., it has been suggested that berberine demonstrates an immunomodulatory impact, augments the cytotoxic properties of NK92-MI cells, and hinders tumour immune evasion through the reduction of PD-L1 expression [61]. Pyrrole alkaloids also exhibit anticancer activity by activating apoptosis, autophagy, and cell necrosis. One of the pyrrole alkaloids (BMA-155CI) was reported to have anticancer potential by modulating apoptosis and autophagy by increasing the expression of various proteins levels, including p65, NF-kB, LC3B, Bax, and Beclin-1 in HepG-2 cell lines [60]. Phenanthroindolizidine alkaloids refer to a class of alkaloids that possess a phenanthrene ring fused with a saturated indolizine ring. Examples of such alkaloids include antofine, tylocrebrine, tylophorine, and tylophorinine. Antofine is a promising phytochemical with potential anti-cancer properties, as it has been observed to induce cell-cycle arrest specifically at the G0/G1 phase. Additionally, it has been found to inhibit the expression of cyclin-dependent kinases (CDKs) and cyclins, specifically cyclins E and D1.

Moreover, antofine also inhibits β-catenin/Tcf transcriptional activity in human colon cancer cells (HCT 116), promoting TNF-α-induced apoptosis [60,62]. Another essential class of organic compounds is steroidal alkaloids (SAs) like solanidine, conanine, etc. These are defined as secondary metabolites that play a significant role in the defense mechanism of plants, with no role in growth, reproduction, and development. The prime mechanism of action of SAs is the activation of apoptotic cell death in a particular cancer cell line, which includes briofilin, which enhances the expression of Bax protein levels, suppresses levels of caspase-3 and Bcl-2 along with segmentation of poly (ADP-ribose) polymerase-1 (PARP-1) in HeLa cells. The biomolecular mechanism underlying the SAs antiproliferative activity might be due to the inhibition of proteins like AKT and MMP-2/9 or by targeting various cell signaling pathways that permits the development of cancer cells [57,63].

### Essential oils and terpenoids

**Figure 5:**
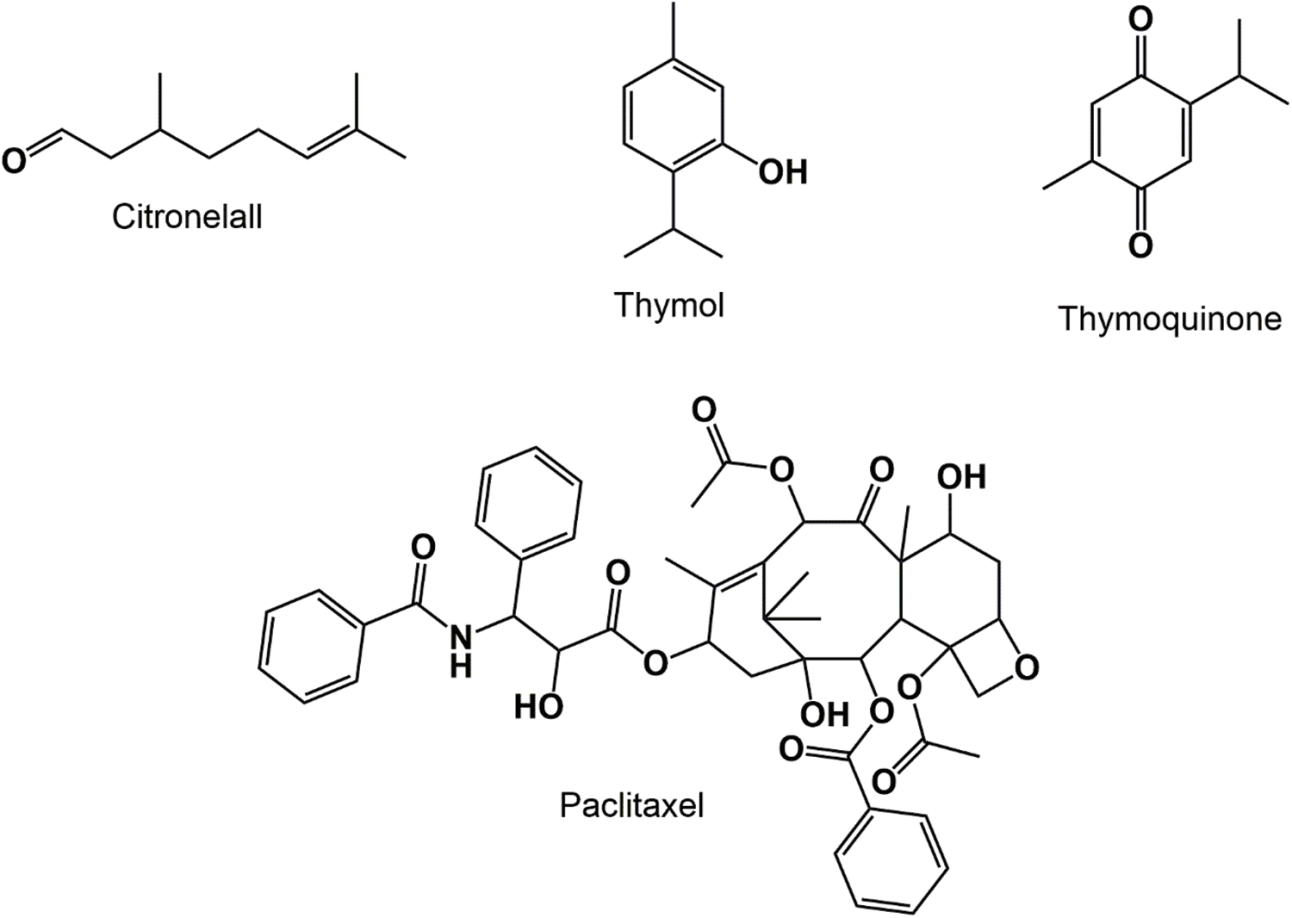
Chemical structures of anti-cancer essential oils and terpenoids.

Essential oils (EOs) and terpenoids (Fig. 5) are multifunctional phytochemicals with a complex structure of plant origin, which have been used for the past several decades because of their biological importance in the treatment and prevention of various diseases. Terpenoids are divided into a variety of categories according on the quantity of isoprene units they contain [64,65]. Thymol, a monoterpene, have been reported to exhibit anti-cancer effect in various type of cancers cell lines like HL-60, and PC3 human prostate cancer cells [66,67]. Research related to the therapeutic efficacy of EOs in anticancer therapy is relatively new [68]. Recently, studies have reported that enantiomer (+)-citronellal EOs obtained from the plant *Corymbia citriodora* and *Cymbopogon nardus*, is also act as a potential microtubule-disrupting agent for the treatment strategy related to cancer [69–71]. Rajiv Gandhi and colleagues reported that chitosan loaded essential oil nanoparticles possess greater cytotoxicity in A549 human lung cancer cell lines compared to simple essential oil treatment [72]. Paclitaxel, a mitotic inhibitor agent, which was originally derived from the bark of the tree *Taxus brevifolia* acts as a potential anticancer agent by inducing mitotic arrest via targeting the cytoskeleton component tubulin, thereby activating the mitotic checkpoints and subsequently leading to apoptosis [65]. Another study from Najjari and colleagues stated that essential-oil loaded nanostructured lipid carriers obtained from the gums of *Pistacia atlantica Desf*. decreases cell viability of SKBR3 breast cancerous cells by inducing cell arrest and apoptosis [73]. Thymoquinone, an EO isolated from *Nigella sativa* has also been discovered to show promising anticancer activity by various mechanisms [74]. A number of studies have been done in order developed an effective novel thymoquinone based phyto-nanophytoformulation, out of which some of the liposomal and polymeric nanoformulation are FDA approved [75–77].

## Influence of Nanotechnology on Phytoconstituents

Phytochemicals generally reach the target site inefficiently due to their low levels of solubility, poor penetration through biological membranes, target specificity, and stability, leading to poor bioavailability in the body [108,109]. This is the main challenge regarding translating phytochemicals’ therapeutic activity to clinical settings [109]. Based on their structural configurations, phytochemicals can be broadly divided into the following classes: alkaloids, terpenoids, flavonoids, and polyphenolic compounds. These various substances, either as substrates, cofactors, or inhibitors of enzymatic reactions, exhibit rich anticancer and cytotoxic activities like phenolic acids (polyphenols); pomolic acid, ursolic acid, oleanolic acid, fomitellic acids, boswellic acids, avicins (triterpenoids); kaempherol, myricetin, quercetin, rutin (flavonoids); and matrine and sanguinarine (alkaloids) as discussed above [110].

The abundance of nanotechnology has a significant impact on phytochemical delivery in many different ways. They have influenced the nanocarrier system from outside as a ligand or delivered to the target site in encapsulated form alone or with synthetic therapeutic agents. Nanosized delivery systems can be organic delivery systems like liposomes and polymeric NPs or inorganic delivery systems like silver, gold, and copper. Phytochemicals could form NPs as a bio-friendly capping agent in green chemistry. Such NPs proved more biocompatible, stable, less toxic, and highly efficient with anti-oxidative properties [108]. In green synthesized nanotechnology, when phytochemicals are entangled or enclosed in the silver NPs have been proven effective for anticancer properties, like the methanolic extract of *Vitex negundo* L., *Acalypha indica*, *Andrographis echioides*, *Premna serratifolia* leaves, *Erythrina indica*, and *Annona squamosa* seed extract. Phytochemical-based metallic NPs impart their anticancer activity by disrupting the lysosome and cell membrane integrity, blebbing, signal transduction, stimulation of signaling pathways, oxidation of lipid and proteins with reactive oxygen species, enhanced mitochondrial activity, with and shrinkage of a nucleus that leads to apoptosis [111]. Anticancer results were analyzed and proved on human hepatoma, Dalton’s lymphoma cells, HeLa cells, and breast cancer cells (MCF-7) with these phytochemicals [110]. Medicinal plants and their components are used in the green synthesis of NPs prominently with silver and gold ions due to their flexible and reliable binding properties to thiol and amine groups, enabling them for surface plasmon resonance (SPR) against cancer [109]. In metallic NPs, iron oxide NPs with seaweed (*Sargassum muticum*), titanium dioxide NPs, cerium oxide NPs, gold-platinum (Au-Pt), and silver–selenium (Ag-Se) bimetallic NPs also showed anticancer activities with quercetin and gallic acid [111]. In a recent study conducted by Pushparaj et al., silver nanoparticles were synthesised using an aqueous extract derived from the leaves of Solanum melongema. The resulting nanoparticles exhibited remarkable efficacy in inhibiting the growth of human MDA-MB-231 breast cancer cells, thus demonstrating promising anticancer activity. Additionally, it demonstrated antibacterial efficacy against various strains including *E. Coli*, *Pseudomonas aeruginosa*, and *Shigella flexneri* etc. This study demonstrates that the utilisation of green synthesis methods for the production of metallic nanoparticles holds promise as a potential strategy for cancer treatment [112].

Such NPs act more efficiently against MCF-7 breast cancer, colon cancer, hepatocellular carcinoma (HepG2 cells), and lung cancer when they have *A. leptopus*, *Cassia tora*, *Gymnema sylvestre* leaf extracts, and *Moringa oleifera* flower aqueous extract, respectively. Apart from NPs, single-walled CNTs (SWCNTs) also represent promising carriers for phenolic acids and Curcumin [2]. For topical delivery, transferosomes transport phytoconstituents like *Curcuma longa* extract, capsaicin, vincristine, and colchicine in a sustained release pattern with more improved bioavailability as they deformed the subcutaneous layer of the epidermis, thereby giving deeper penetration [111]. Another advancement in anticancer chemotherapy is the combination of phytoconstituents with cytotoxic agents that the National Cancer Institute (USA) encouraged to combat multidrug resistance (MDR) undesirable side effects by using the potential antitumor activities of plant extracts.

## Various Types of Phyto-Based Nanoformulations

## Vesicular Systems

### Liposome

Liposome is a microscopic lipid vesicle consisting of an inner aqueous core and an outer bulk separated by a thin lipid bilayer. Liposomes are estimated to range from 10 nm to 1 m or more significant. The inner aqueous core facilitates the targeted delivery of vaccines, enzymes, proteins, and drugs to the intended site. Stealth liposomes are superior to transferosomes in site-specific targeting and delivering drugs across rigid barriers. Besides their biocompatibility, biodegradability, and amphiphilicity, liposomes can encapsulate polar and nonpolar therapeutics [113]. A mucoadhesive polymer can be applied to the surface of liposomes to deliver targeted drugs, or liposomes can be ligated to facilitate drug delivery. The work by Shu et al. aimed to treat myelogenous leukemia by employing liposomes laden with betulinic acid to target HepG2 cells. Mannosyl erythritol lipid-A (MEL-A) was added to the surface of liposomes during their creation in order to improve transfection effectiveness and make it easier for DNA to bind to liposomes. Surface-modified betulinic acid liposomes with MEL-A augment the cross-way permeation of these liposomes into the cells. This ultimately paves the way for faster cell apoptosis. In addition to enhanced anticancer activity, the G1 phase blockage was also responsible [114].

Likewise, curcumin nanoliposomal delivery shows advanced antitumor potential. Curcumin nanoliposome formulations (PG-LipCUR and LipCUR) actively eliminated breast cancer cells (TUBO and 4T1). Compared to free CUR, liposomal curcumin has a longer half-life based on the pharmacokinetic study. Because of the fusogenic properties of the lipids used, in the biodistribution study in mice, both nano liposomal formulations showed more significant tumor accumulation than pure curcumin [115]. Differential nano-formulation approaches have emerged to address these challenges. As a result of these studies, Greil et al. investigated the efficacy of increasing liposomal curcumin doses (Lipocurc TM) in metastatic cancer patients. During phase I trials, over 32 patients were treated with liposomal curcumin, with a dose of 300 mg/m^2^ liposomal curcumin over 6 h reported as the most tolerated dose by patients [116]. These lipocurcumin formulations were proven harmless, and ceramide generated upon pretreatment significantly alleviated cancer markers through apoptotic and cell-cycle arrest phenomena.

Further, in contrast to the parent curcumin formulation, the liposomal formulation showed a significantly higher dose-effect profile with minimal harm to the patients. However, LipocurcTM contains curcumin polyphenols, which include DMPC (2:0-1, 2-dimyristoyl-sn-glycero-3-phosphocholine) and DMPG (2:0-1, 2-dimyristoyl-sn-glycero-3-phosphorylglycerol), which protect against cancer. Patients with cancer experienced a significant increase in curcumin plasma levels after receiving the liposomal carrier intravenously. Consequently, the excretion rate of the drug and half-life are also lower than those of physically well-being individuals upon dose withdrawal. In a phase-I study using liposomal curcumin in metastatic tumor patients, they obtained steady plasma concentrations with a substantial but brief reduction in tumor markers, including CEA (Carcinoembryonic antigen), PSA (prostate-specific antigen), and CA 19–9 (cancer antigen). Additionally, the sphingosine kinase inhibitory effect of liposomal curcumin implies that it may be used alone or in combination with other cancer treatments to lessen the severity and frequency of cancer recurrences [117]. AbouSamra et al. formulated rutin loaded liposomes for the treatment of hepatocellular carcinoma. It was observed that developed formulation effectively enhanced the solubility and biological activity of rutin. Moreover, to prepare the desirable liposomes, β-sitosterol was used as an alternative to cholesterol and thus, termed it as “Phyto-stereosomes”. This study provides evidence supporting the potential therapeutic efficacy of nano-encapsulated Rutin within this novel vesicular nanoformulation designed to promote its action against hepatocellular carcinoma [118].

### Niosomes

An innovative drug delivery system, niosomes use nonionic surfactants to deliver therapeutics. Surfactant molecules are two-fold in structure, which encourages them to form a bilayer structure. Two surfactant layers are formed by hydrophilic and hydrophobic ends arranged in external and internal locations. In a unique carrier, two kinds of drugs are encapsulated by this lamellar morphology. Several factors include surfactants, cholesterol levels, critical packing parameters, drugs used, and shape niosomes are need to be optimized for efficient drug delivery. For drug delivery, these systems can be designed to be administered orally, parenterally, topically, and so on. Although liposomes resemble niosomes in structure, niosomes are more stable and cost-effective than liposomes. Unlike ions, niosomes remain in the bloodstream for quite some time, making them an excellent drug-delivery vehicle [119]. Zare-Zardini et al. evaluated the *in vitro* test on Ginsenoside Rh2- loaded niosomes and prepared two formulations: 5% DOTAP-loaded niosomal formulation and DOTAP-free niosomal formulation. Additionally, cholesterol and Span 60 were included in niosomal formulations, and DOTAP was used as cationic lipid. However, DOTAP-loaded nano-niosomal formulations containing Ginsenoside Rh2 had average size, PDI, zeta potential, and encapsulation efficiency of 93.5 ± 2.1 nm, 0.203 ± 0.01, + 4.65 ± 0.65 mV, and 98.32% ± 2.4%, respectively. It was found that niosomal vesicles have a round shape and smooth surfaces. Niosome release of Ginsenoside Rh2 followed a biphasic pattern. It was demonstrated that adding DOTAP to niosomal formulations enhanced cellular uptake compared to DOTAP-free niosomal formulations. Furthermore, it also improved cellular uptake and cytotoxic activity in PC3 cells [120]. Barani and team designed and evaluated Carum-loaded Niosomes targeting breast cancer. A chemical compound known as thymoquinone (TQ) is present in the Carum carvil (C. carvil) seeds. TQ has many applications in medicine, including cancer therapy. Although TQ is hydrophobic, it is poorly soluble, permeable, and bioavailable in biological mediums. Hence, it was designed to minimize these drawbacks by combining Ergosterol (herbal lipid) with *Carum carvil* extract (Carum) and non-ionic surfactants. However, two formulations of noisome containing TQ and Carum was prepared and evaluated. A niosome loaded with TQ and *Carum* was showed an entrapment efficacy such as 92.32% ± 2.32% and 86.25% ± 1.85, respectively. As compared with free TQ, both formulations provided a controlled release. Loaded niosomes exhibit more significant anticancer activity against MCF-7 cancer cells, according to the results of the MTT experiment and a flow cytometric study. Interestingly, Nio/TQ, Nio/Carum, and TQ formulations showed arrest in the G2/M cell cycle and a decline in the migration of MCF7 cells. TQ and Carum-loaded niosomes illustrated appreciable encapsulating power for poorly soluble phytoconstituents and could be utilized as a carrier to enhance site-specific drug targeting in breast cancer treatment [121].

### Ethosomes

Ethosomes are elastic NPs based on phospholipids. The ethosomal system primarily comprises phospholipids, water, and high ethanol concentrations (20%–45%). Among the benefits of ethanol is that it enhances skin penetration and is a rich permeation enhancer [122,123]. Since ethosomes have improved skin permeability, they can be used as drug nanocarriers to treat skin cancer. The ethanol composition may penetrate deeper into the skin because it provides the vesicles with the characteristics of soft elastic nature. Due to its ability to affect the two-layered structure of stratum corneum, ethanol may enhance drug penetration in phospholipid vesicles [124]. Moreover, recovering the fluidity of the lipid membrane of the skin. A unique characteristic of ethosomes is that they act as a shield from the outer environment, simultaneously helping move the drug across the different layers of skin and timely releasing the drug [125–127]. In this way, skin cancer treatment was expected to benefit from effective drug delivery. Furthermore, Lin et al. investigated the effects of ethosomes enclosing evodiamine (EVO) and Berberine chloride (BBR) [128]. It aimed to develop a novel method of single-step injection for topical anti-melanoma therapy. Propylene glycol (PG) cholesterol and soybean lecithin (SPC) cholesterol are formed in different amounts. Ethosome particle size increases as SPC (180 mg) increases (227 ± 6 nm). However, EVO and BBR were entrapped more than 90% efficiently by the ethosomes formulation. The *in vitro* findings verified that the formulation (Ethosome/BBR-EVO) reached the epidermis layer of the skin. They also enhance penetration as well as drug delivery. Overall, the formulation was effective in drug delivery against skin cancer [128]. According to the studies discussed above, PTX (Paclitaxel) was found extended drug release to the target when loaded into ethosome [129]. In addition, curcumin-loaded ethosomes were compared with PTX-loaded ethosomes developed in-house. Curcumin-loaded ethosome deposited more than 60% of the drug on the skin, while PTX-loaded ethosome deposited less than 60% [130,131]. The release profile of CUR gel and PTX gel was 12 and 16 h, respectively. Nevertheless, the prepared ethosomal gel needed to be studied pre-clinically and clinically. It can be concluded that ethosome is an effective system for vesicular drug delivery. In this way, the drug is loaded more efficiently into the skin and the permeability of the drug is increased. Furthermore, ethosomes have a high zeta potential, which indicates independent repulsion, thereby preventing aggregation or fusion due to electrostatic interactions.

### Nanomicelles

A micelle is a nanoscale material formed from the aggregation of amphiphilic molecules in an aqueous environment above the critical micellar concentration. In most cases, micelles consist of an amphiphilic block copolymer and a core-shell structure. Nanomicelles possess unique properties, potentially holding water-soluble and insoluble solutes [132]. The nanocarriers were highly influential in loading chemotherapeutics explicitly formulated to target ovarian cancer sites [133,134]. A micelle with a size between 10 and 100 nm is more porous, more likely to be endocytosed by ovarian cancer cells, and less likely to target normal cells [135]. Among nano micelles, remarkable properties are *in vivo* stability, enhanced biocompatibility, sustained plasma circulation, perforation of inflammation, and incorporation of hydrophobic chemotherapeutics. Previous reports described using immunotherapy against melanoma based on lipid-based Trp2 peptide vaccines by Lu and his team. In tumors, suppressive immune microenvironments hinder vaccine therapies. Using curcumin (CUR) as a vaccine enhancer will remodel the tumor microenvironment to enhance its effectiveness. CUR-PEG (polyethylene glycol), a curcumin micelle, was administered intravenously to treat the tumor. Vaccine treatment and CUR-PEG treatment combined had a synergistic antitumor effect in B16F10 mice compared to their personal effects. According to *in vivo* data, the combination therapy significantly increased CTL (Cytotoxic T lymphocyte) responses, found 41.0 percent more or less than 5% for specific killing, and IFN-γ production resulted in a 7-fold increase. The combination therapy, including MDSCs and Treg cells, IL-6, and CCL2, significantly decreased several immunosuppressive factors. There was also a significant increase in pro-inflammatory cytokines, including TNF and IFN (interferons (IFNs) and tumor necrosis factor (TNF), as well as CD8+ T cells, as a result of the combination therapy. Results showed that tumors were switching from M2 to M1 phenotypes. CUR-PEG and vaccine significantly decrease the STAT3 activity (76%). Lu et al. suggested that CUR-PEG might be promising for improving immune therapy for advanced melanoma [136]. Patra and the team formulated a blend of polymeric micelles utilizing quercetin (QCT) to target different cancer types. Indeed, QCT, a flavonoid, is an excellent source of antioxidants that exhibit cancer-protective and anti-proliferative properties. However, due to their water-insoluble nature, they are less active. Therefore, the mixed polymeric micelles (MPMs) method and their physicochemical properties were examined. These MPMs demonstrated anticancer activity in ovarian (SKOV-3 and NCI/ADR, epithelial and multi-drug resistant cell lines, respectively) and breast cancer (MCF-7 and MDA-MB-231). According to *in vitro* experiments, MPMs released QCT more slowly than free QCT. When compared with pure QCT, QCT had a markedly improved solubility. Due to their low critical micelle concentration, MPMs have high stability in aqueous media. In all cancer cell lines, the concentration that inhibited 50% growth (IC_50_) was significantly lower for both micellar preparations than free QCT. There is potential for further development of MPMs containing QCT, which may be effective for treating cancer types ranging from cancerous to non-cancerous [137].

**Figure 6:**
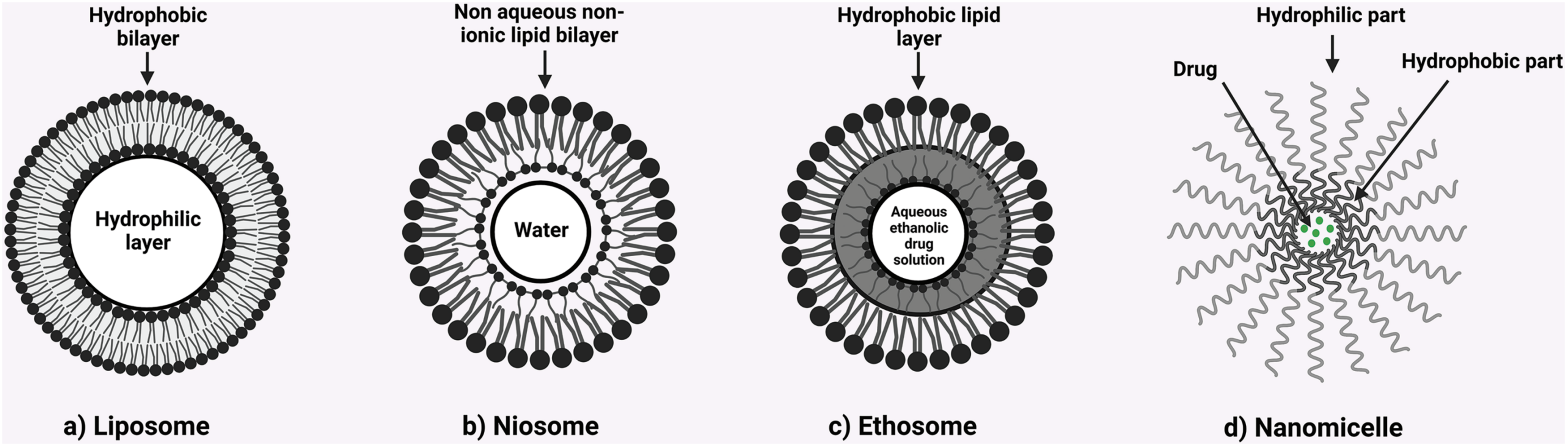
Illustration representing various vesicular systems for the delivery of phyto-constituents

Fig. 6 provides an illustration of above discussed vesicular systems for the delivery of phytocostituents.

## Particulate Systems

### Polymeric nanoparticles

Polymeric NPs are 1 to 100-nm-sized particles typically loaded with active compounds entrapped inside or surface-adsorbed onto the polymer core. NPs are classified as either nanocapsules or nanospheres, depending on their morphology. Several diseases can be treated with polymeric NPs for targeted drug delivery. As drug carriers, polymeric NPs have several advantages. They can control the drug release from the reservoir, protect drugs and other molecules from the environment, and improve their bioavailability and therapeutic index [113,114,116,138]. An herbal-based nano-formulation delivers targeted drug delivery to minimize the side effects of anti-cancer drugs and conventional drug delivery systems. Hence, *A. absinthium* extract-loaded polymeric NPs (NVA-AA) were formulated and evaluated against breast cancer (MCF-7 and MDA MB-231), also focused on a protein that targets cytotoxicity [139].

Three monomers were co-polymerized to create poly-meric NPs in the preparation, namely N-vinylpyrrolidone-acrylic acid (NIPAAM), N-vinylpyrrolidone (VP) and acrylic acid (AA). Synthesized NPs had a size of 131.4 and 19.7 nm, and the PDI was 0.1 ± 0.03 at 25 degree Celsius. As a result of TEM analysis (*Transmission electron microscopy*), the synthesized NPs had a spherical shape and were highly monodispersed, having an average size of 110 nm × 12.6 nm. However, the IR spectra showed broad, intense peaks at 3280.35 and 1636.78 cm^−1^. As a result, it confirmed the polymerization of NIPAAM with VP and AA. Against MCF-7 and MDA MB-232, whole plant extract exhibited more significant cytotoxicity than different parts of the plant (with lower IC_50_ values of 307.16 × 20.4 g/mL and 338.55 × 15.7 g/mL, respectively). To conduct further research, the formulation found an entrapment efficiency of 84.8% and a loading capacity of 21.2%. LCMS/MS and STRING analysis identified the protein targets and their interactions, validated for using qPCR and BLI. The LCMS/MS analysis demonstrated that the cytotoxicity was caused by modifying protein molecules implicated in vesicular transport, apoptosis, proliferation, and metastasis. Additionally, vesicular trafficking and apoptosis networking are connected by UBA52 in MCF-7 and TIAL1, PPP1CC in MDA MB-231 cells, producing effective results against breast cancer [139]. Fang, and team designed charge-reversing NPs that could effectively target the cells’ mitochondria while responsive to different pH environments. A comparison was made between vitamin B6-oligomeric hyaluronic acid-di-thiodipropionic acid-berberine/curcumin (B6-oHASS-Ber/CUR) and vitamin B6-oligomeric hyaluronic acid-di-thiodipropionic acid-berberine/curcumin (oHA-SS-Ber/CUR). The lipophilic cation Berberine (Ber) was conjugated with oligomeric hyaluronic acid (oHA-SS-Ber) through disulfide bonds. As a result of conjugating B6 with oHA, B6-oHA-SS-Ber was synthesized using the dialysis method and produced two types of Cur-loaded NPs (CUR-NPs). In tumor tissue acidic microenvironments, B6 pKa permits its charge to move from a negative to a positive one, making it more effective at targeting mitochondria. In transmission electron microscopy (TEM), B6-oHA-SS-Ber/Cur micelles could self-assemble in water and form spherical NPs with a hydrodynamic diameter of 172.9 ± 13 nm. Studies comparing oHA-SS-Ber/Cur micelles with B6-oHA-SS-Ber/Cur micelles showed that B6-oHA-SS-Ber/Cur micelles showed better cytotoxicity, lysosome escape, mitochondrial distribution, and cellular uptake. Micelles containing B6-oHA-SS-Ber/Cur exhibited effective effects on tumour growth *in vivo* [140].

### Solid lipid nanoparticles (SLNs)

As alternatives to the traditional colloidal delivery systems, the SLNs are promising in providing a blend of NPs and liposomal systems while being relatively non-toxic. Biocompatible lipids are used in SLN to provide a highly tolerable safety profile. Drug and gene delivery can be enhanced by integrating SLNs with hydrophilic, lipophilic drugs, proteins, and nucleic acids [141]. Akanda et al. designed transferrin (Tf)-conjugated solid lipid NPs (Tf-SLNs) loaded with curcumin for active prostate cancer cell targeting. Cancer mortality from prostate cancer is one of the leading causes of death in men worldwide and one of the most challenging to treat. However, *in vitro* unloaded Tf-SLNs demonstrated less cytotoxicity, while Tf-Cur-SLNs exhibited effective anti-proliferative action. Furthermore, Tf-Cur-SLNs showed a significant increase in cellular uptake (*p* < 0.05/=0.01) compared to unconjugated SLNs. While compared to Cur-SLNs and Cur solution, bioconjugated Tf-Cur-SLNs also improved in primary and late apoptotic populations. In contrast to the control group, the Tf-Cur-SLNs significantly reduced the size of tumors in mice bearing prostate cancer (392.64 mm^3^ after 4 weeks, *p* < 0.001). SLNs bio-conjugated with bioactive molecules could potentially cure cancer *in vivo*, based on the findings of this study [142]. In another study, researchers developed Silibinin loaded (SLNs) for targeting lung cancer. According to the results, the developed formulation indicated that they are suitable for inhalation. The Silibinin loaded- SLNs were effective against A549 cells, based on *in vitro* cellular efficacy. Inhalable SLNs that could be given to the lungs without complication were successfully introduced with this method [143]. Farhadi et. al., fabricated SLNs loaded with chrysin and subsequently functionalized the nanoparticles by conjugating them with folate-bound chitosan. The prepared demonstrated inhibitory effects on PANC, MCF-7, A2780, and HepG2 cell lines, which are known to be malignant. Furthermore, the prepared formulation exhibited the ability to scavenge free radicals, while also demonstrating the capability to inhibit angiogenesis [144]. Therefore, it can be regarded as a potentially auspicious candidate for preclinical and clinical investigations in the context of pancreatic cancer.

### Gold NPs

Gold NPs have the potential to act as drug carriers in recognition of their optical, SPR, and tunable properties. They have an extensively broader size range available from 1 to 150 nm. Gold NPs are easily adjustable due to their negative charge and can efficiently hold ligands, drugs and other biomasses. Furthermore, gold NPs are practically safer, biocompatible non-reactant materials that are becoming preferable as drug-carriers [145,146]. Lee interestingly encapsulated gold NPs in chitosan and designated spherical, star and rod shapes to these NPs for understanding the effects of different shapes on cytotoxic potential and cellular uptake of gold NPs. The reduction of gold salts into gold nanospheres was achieved through green tea extract. To prepare gold nanostars, as-prepared nanosphere solutions were used as seed solutions. A conventional method was used to synthesize gold nanorods. According to FTIR studies, the −OH functional groups are formed when polyphenols are oxidized to C=O while Au salts are reduced to AuNPs. The presence of C=O functional groups in nanospheres has demonstrated oxidation of –OH functional groups during synthesis, as demonstrated by their findings. A UV-visible spectrophotometry study revealed that all three gold NP types (nanospheres, nanostars, and nanorods) have characteristic surface plasmon resonance bands, with nanospheres showing 537 nm, nanostars 600–800 nm and nanorods 797 nm, based on zeta potential of 190.7 nm, nanostars 123.9 nm, and nanorods 33.23 nm. Accordingly, the increase in zeta potentials for the nanospheres and nanostars indicates successful capping with chitosan.

**Figure 7:**
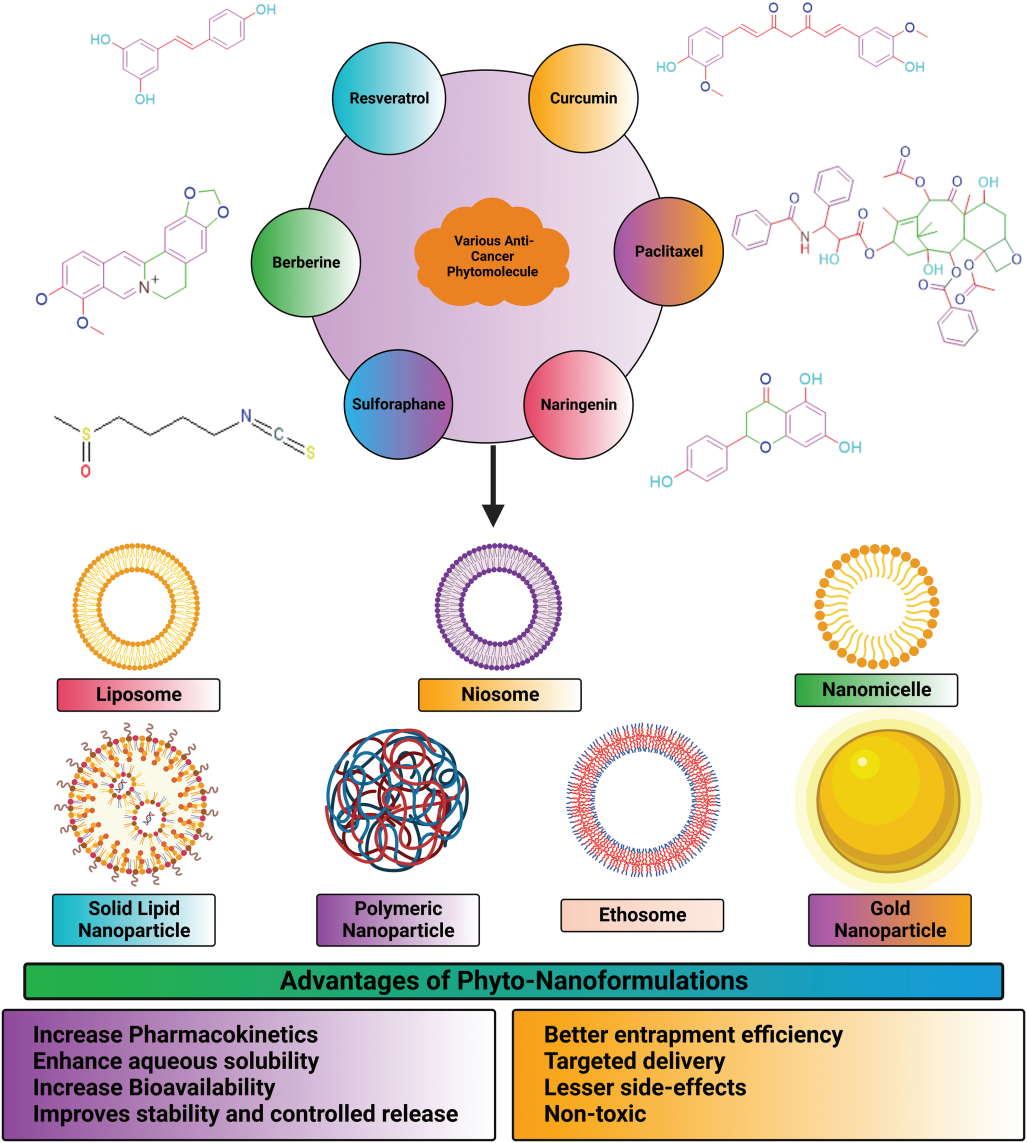
Advantages of nano-encapsulated phyto-nanoformulations in treatment of cancer.

Additionally, the nanorods’ zeta potential was increased, suggesting that their surfaces were capped with chitosan. The lattice structures in all three shapes were visible in high-resolution transmission electron microscopy images, indicating that the NPs are crystalline. Typical particle dimensions of nanospheres were 8.7 nm × 1.7 nm, nanostars were 99.0 nm × 47.0 nm, and nanorods were 60.4 nm × 16.4 nm. Among nanorods, nanostars, and nanospheres, nanorods had the highest cytotoxicity. The human hepatocyte carcinoma cells (HepG2) have displayed varied cellular update capacities of gold NPs ranging from the highest uptake by nanospheres to the slightly lower uptake by nanorods to the lowest being with the nanostars [147]. This study has provided a different perspective on selecting the appropriate shapes of gold NPs for achieving better therapeutic profiles of the loaded drugs. Lupeng Li and colleagues developed gold NPs of *Marsdenia tenacissima* (MT) for targeting liver cancer HepG2 cells. For thousands of years, *Marsdenia tenacissima* has been used in Chinese medicine to treat respiratory problems, asthma, and rheumatism. The FTIR analysis shows that the MT-AuNPs show peaks at 3331.43 and 1637.27 cm^−1^, corresponding to the O–H and C=C stretching modes. MT-AuNPs at 59.62 × 4.37 g were found to be cytotoxic after 24 h. The migration assay of MT-AuNPs confirmed the induction of apoptosis, changes in MMP and generation of ROS. Up-regulation of apoptotic proteins (Bax, caspase-9 and caspase-3) confirmed the apoptosis phenomenon, and further this was also affirmed by the down-regulation of anti-apoptotic proteins (Bcl-2 and Bcl-XL). The anti-cancer profile of MT-AuNPs against liver cancer was established through this study [97]. Fig. 7 represents advantages of nano-encapsulated phyto-nanoformulations in treatment of cancer.

## Clinical Development, and Regulatory Concerns Associated with Anticancer Phytoconstituent’s Herbal Nanoformulations

Various medicinal plants in clinical trials show positive results for their anticancer efficacy. Two approaches are recommended to the sponsors to prove that variations in different batches will have no effect on the clinical response of the botanical medicinal product. The initial approach involves the identification of batch effects on clinical endpoints through the utilisation of multiple-batch analyses. This method allows for the assessment of potential heterogeneity in clinical outcomes, such as subgroup analysis. The second approach, on the other hand, demonstrates that clinical response is not influenced by dosage. It investigates the effectiveness of various doses in comparison to placebo or control, and determines whether they are either superior to placebo/control or non-inferior to active treatment. This demonstrated that as long as batches were regulated within the bounds of specification, the hereditary heterogeneity of chemical components could not effect final clinical outcomes. These days, combinatorial therapy is used with phytocompounds in conjugation with other chemopreventive agents against single drugs to combat the side effects or achieve higher sensitivity or synergistic or additive effects. Multiple-herb preparations (e.g., Shen qu) are also becoming part of the botanical combination drug. The beta error (wrong negative finding) increases with the number of botanicals evaluated in a factorial trial. A five-arm trial (or six if a placebo group is included) comparing ABCDE against ABCD, ABCE, ABDE, and BCDE was presented in the introduction to the proposed updated Combination regulations (21CFR300.50). The granting of waivers by the Agency is permissible under the proposed combination rule in cases where it is impractical to conduct a comprehensive analysis of all constituent elements. If a combination contains multiple active substances without the deliberate intention to combine them, it would not typically be classified as a fixed dose combination under the proposed Fixed-Combination regulations. Additionally, the revised 21 CFR 300.60 suggests that a waiver may be granted under the proposed rule in such cases.

So, it becomes an imperative research area for pharmaceutical formulators and biomedical scientists to integrate green nanotechnology into cancer science for the formulation of superior plant-based antineoplastic agents. Many FDA-approved phytoconstituents with their clinical trials are listed below in Tables 2 and 3.

**Table 2:** List of Phytochemicals under clinical trials and in the market for clinical practice

S. No.	Phytochemical	Plant source	Indication	Clinical trial No.	Estimated/Study completion date (month/year)	Reference
1.	Alvocidib [Flavopiridol]	*Dysoxylum binectariferum* (Meliaceae)	Chronic lymphocytic leukemia (CLL)	NCT00003256 NCT00445341 NCT00058240	4/2004 10/2012 2/2009	[150]
2.	Topotecan [Hycamtin]	*Camptotheca acuminata* Decne (Nyssaceae)	Lung cancer	NCT00611468 NCT00382733 NCT01492673	8/2009 3/2011 10/2018	[151]
3.	Belotecan hydrochloride	*Camptotheca acuminata* Decne (Nyssaceae)	Epithelial ovarian cancer, Small cell lung cancer	NCT00430144 NCT01497873 NCT01630018	10/2010 3/2018 6/2014	[152]
4.	Irinotecan HCl	*Camptotheca acuminata* Decne (Nyssaceae)	Metastatic pancreatic cancer	NCT01114555 NCT01220063 NCT00003748	11/2018 8/2018 1/2005	[153]
5.	Gimatecan	*Camptotheca acuminate* (Nyssaceae)	Pancreatic cancer	NCT00410358 NCT00032903 NCT04029909	2/2008 10/2005 12/2022	[154]
6.	9-amino-20(S)- camptothecin [9-amino-CPT]	*Camptotheca acuminate* (Nyssaceae)	Ovarian cancer	NCT00002635 NCT00003551 NCT00002671	4/2000 9/2004 2/2001	[155]
7.	AR-67 [10-Hydroxy Camptothecin]	*Camptotheca acuminate* (Nyssaceae)	Breast cancer	NCT00956787 NCT01202370 NCT00389480	12/2014 7/2011 5/2009	[109]
8.	Karenitecin [Cositecan]	*Camptotheca acuminate* (Nyssaceae)	Metastatic melanoma	NCT00010218 NCT00062478 NCT00477282	1/2006 8/2002 6/2013	[156]
9.	Exatecan mesylate anhydrous	*Camptotheca acuminate* (Nyssaceae)	Sarcoma, Lung cancer	NCT00045318 NCT00055939 NCT00005091	10/2007 4/2006 8/2003	[157]
10.	BN80927 [Elomotecan]	*Camptotheca acuminate* (Nyssaceae)	Advanced malignant solid tumors	NCT01435096	10/2007	[158]
11.	Namitecan [ST-1968]	*Camptotheca acuminate* (Nyssaceae)	Solid malignancies	NCT01748019	12/2011	[159]
12.	5-(2′- Hydroxyethoxy)-20 (S)-camptothecin [DRF-1042]	*Camptotheca acuminate* (Nyssaceae)	Solid tumor patients	NA	----	[160]
13.	Vincristine sulfate [Kyocristine]	*Vinca rosea* Linn. (Apocynaceae)	Acute Lymphoblastic Leukemia	NCT00022555 NCT00516295	7/2003 1/2010	[161]
14.	Vinorelbine tartrate	*Vinca rosea* (Apocynaceae)	Melanoma	NCT00432562 NCT00675597 NCT01222715	12/2007 2/2012 6/2015	[162]
15.	Fosbretabulin tromethamine [CA4P]	*Combretum caffrum* (Combretaceae)	Liver cancer	NCT01305213 NCT02132468 NCT00653939	11/2016 8/2016 10/2011	[163]
16.	Ombrabulin [AVE8062]	*Combretum caffrum* (Combretaceae)	Neoplasms, Malignant	NCT00699517 NCT01907685 NCT01263886	4/2013 2/2011 10/2012	[164]
17.	Idronoxil [Phenoxodiol]	*Glycine max* Linn. (Fabaceae)	Prostate cancer, Fallopian tube cancer	NCT00382811 NCT00557037 NCT00382811	4/2011 11/2009 4/l2011	[165]
18.	Genistein	*Glycine max* Linn. (Fabaceae)	Colon cancer, Rectal cancer, Colorectal cancer	NCT01985763 NCT01325311 NCT00244933	10/2018 7/2014 10/2009	[166]
19.	Ingenolmebutate [PEP 005]	*Euphorbia peplus* (Euphorbiaceae)	Actinic keratosis	NCT03569345 NCT00108121 NCT02723721	5/2018 5/2006 11/2020	[167]
20.	Betulinic acid	*Betula pubescens* (Betulaceae)	Anticancer	NCT00701987	6/2009	[168]
21.	Maytansine	*Maytenusovatus* (Celastraceae)	Diffuse large b-cell lymphoma, Breast cancer	NCT02289833 NCT00934856 NCT01641939	8/2018 10/2013 4/2016	[169]
22.	Sulforaphane	*Brassica oleracea* (Brassicaceae)	Breast cancer	NCT03232138 NCT01228084 NCT02970682	4/2016 5/2013 3/2019	[170]
23.	Epigallocatechin gallate [EGCG]	*Camellia sinensis* (Theaceae)	Uterine fibroids	NCT02891538 NCT02577393 NCT00459407	6/2024 8/2022 8/2010	[171]
24.	Curcumin	*Curcuma longa* (Zingiberaceae)	Prostate cancer	NCT01917890 NCT01160302 NCT02554344	10/2013 1/2016 1/2017	[172]
25.	R-(-)-Gossypol acetic acid	*Gossypium herbaceum* (Malvaceae)	Extensive stage small cell lung cancer	NCT00773955 NCT00397293 NCT00666666	8/2010 12/2008 6/2012	[173]
26.	Beta-lapachone [ARQ 501]	*Tabebuia avellanedae* (Bignoniaceae)	Advanced solid tumors	NCT00524524 NCT00099190 NCT00358930	8/2008 11/2006 8/2007	[174]
27.	Cryptophycins 52 [LY355703]	*Cyanobacteria, Nostoc* sp. [ATCC 53,789,Nostoc sp. GSV 224] (Nostocaceae)	Hematologic tumor	NA	---	[175]
28.	Lovastatin	*Pleurotus ostreatus* (Pleurotaceae)	Gastric cancer	NCT00462280 NCT00584012 NCT00902668	2/2012 4/2009 4/2013	[176]
29.	Kanglaite [Coix Seed Oil]	*Coixlacryma-jobi* (Cramineae)	Cancer cachexia	NCT02553187 NCT03986528 NCT00733850	7/2018 5/2022 6/2014	[177]
30.	Simvastatin	*Aspergillus terreus* (Trichocomaceae)	Breast cancer stage IV, Small cell lung cancer	NCT03324425 NCT00334542 NCT00944463	12/2030 11/2011 2/2014	[178]
31.	Paclitaxel [Taxol]	*Taxus brevifolia* (Taxaceae)	Breast neoplasm	NCT03096418	8/2022	[179]
32.	Taxoprexin [DHA-paclitaxel]	*Taxus brevifolia* (Taxaceae)	Breast tumors	NCT00244816 NCT00249262 NCT00243867	4/2007 4/2007 8/2008	[180]
33.	Larotaxel [XRP 9881]	*Taxus baccata* L. (Taxaceae)	Breast cancer	NCT00387907 NCT00417209 NCT00625664	3/2008 11/2009 2/2011	[181]
34.	Ortataxel [IDN- 5109/BAY 59–8862]	*Taxus baccata* L. (Taxaceae)	Carcinoma, Renal cell	NCT01989884 NCT00054314 NCT00039169	12/2016 4/2003 12/2001	[182]
35.	MAC 321 [Milataxel]	*Taxus baccata* L. (Taxaceae)	Carcinoma, Non-Small-cell lung	NCT00685204 NCT00063219 NCT00063427	5/2008 2/2005 2/2004	[183]
36.	UNII-A2VM2V569A [TPI-287]	*Taxus baccata* L. (Taxaceae)	Neuroblastoma, Medulloblastoma, Relapse	NCT00867568	2/2016	[184]
37.	BMS-275,183 [Oral Taxane]	*Taxus baccata* L. (Taxaceae)	Non-small cell lung cancer	NCT00103831	5/2005	[185]
38.	DJ 927 [Tesetaxel]	*Taxus baccata* L. (Taxaceae)	Advanced melanoma, Cancer	NCT01064713 NCT00077077 NCT01573468	10/2014 1/2006 8/2014	[186]
39.	Cabazitaxel [Jevtana]	*Taxus brevifolia* Nutt. (Taxaceae)	Colorectal cancer	NCT01751308 NCT01438307 NCT01308580	2/2016 9/2015 8/2015	[187]
40.	Elliptinium acetate [Celiptium]	*Bleekeria vitensis* (Apocynaceae)	Anti-tumor	NA	---	[188]
41.	Omacetaxine mepesuccinate [Synribo]	*Cephalotaxus harringtonia var. drupacea* (Cephalotaxaceae)	Myeloid Leukemia, Chronic	NCT00006364 NCT00462943 NCT00675350	9/2005 6/2013 1/2009	[189]

**Table III:** List of marketed phytochemical-based Nanoformulations administered by I.V

S. No.	Brand Name	Manufactured by	Phytochemicals	Type of Nano-formulation	Type of cancer
1.	Abraxane®	Bristol Myers Squib, USA	Paclitaxel	Albumin-NPs	Breast, lung and Pancreatic cancer
2.	Onivyde®	Ipsen Biopharmaceuticals, Inc., USA	Irinotecan	Irinotecan-Hcl liposome	Pancreatic and Advanced breast cancer, Refractory pediatric malignancies, NSCLC
3.	Navelbine/ NanoVNB®	Pierre Fabre Pharmaceuticals, Inc., France	Vinorelbine tartrate	Liposome	Non-small cell lung, Breast and Ovarian cancer
4.	Marqibo®	Talon Therapeutics, Inc, South San Francisco, CA, USA	Vincristine-sulfate	Liposome	Acute lymphoblastic leukemia (ALL)
5.	IMX-110	Immix Biopharma, Los Angeles, CA, USA	Curcumin	Combined therapy: Curcumin/ doxorubicin encapsulating NPs	Advanced solid tumors
6.	DoceAqualip	Intas Pharmaceuticals Ltd., Chinubhai Centre, Ashram Road, Ahmedabad, India	Docetaxel	Nanosomal lipid suspension	Solid tumor, Prostate and Breast cancer, Squamous cell carcinoma of the head and neck, Gastric adenocarcinoma

Any regulatory agency viz. The food and Drug Administration (FDA) and European Medicines Agency (EMA) approves any drug for marketing authorization when it shows significant benefits, statistically outweigh the risk results through all phases of its clinical trials. Although plant-based compounds are considered safer and less toxic than synthetic compounds, but to avoid any unregulated use against different diseases much evidence of their side effects must be reported [148]. Regulation of herbal constituents is challenging as herbal drug’s medicinal constituent profiles generally vary when the same plant species grown in different seasons, processing, growing/cultivation conditions (e.g., soil, light, temperature, humidity, and time of harvesting), differences in plant genetics that leads to variation in chemical composition and biopharmacological activity of botanical raw materials and ultimately standardization of quality, safety and efficacy affected due to lack of data. Therefore, conventional chemistry, manufacture and control (CMC) approaches used for quality control (QC) of small molecules are sometimes not sufficient. It is recommended that sponsors of INDs (Investigational New Drugs) should establish and execute Good Agricultural and Collection Practises (GACP) in accordance with the WHO Guidelines on GACP20 or the EMA Guideline on GACP for Starting Materials of Herbal Origin21. These practices should be implemented at the grower or farm levels to provide guidance for the production of botanical raw materials in specific geographical regions [149]. To ensure batch-to-batch consistency in herbal anticancer drug’s a “totality-of-the-evidence” approach should be adopted that includes chemical identification, fingerprint analysis, quantification of active or chemical constituents, and a biological assay with nonclinical studies and clinical effectiveness showing no interaction with the clinical batches [149].

Another challenge is harmonizing the marketing authorization of herbal anticancer drug among different regulatory agencies. For this specific efforts are taken by the FDA by seeking responses in questionnaires against their guidelines. Now, it is the need of the hour that for standardized and high-quality production of plants with a uniform metabolite profile, we have to focus on biotechnical approaches and genetic studies that declare safe or unsafe once and for all. Next challenge is that unfortunately, all the developed and approved phytoconstituent-based pre-parations are intravenously or subcutaneously administered. The oral route is the most convenient and preferred, so in this connection, research is required to overcome the solubility, acidic and enzymatic degradation, and first-pass effect. The next big challenge is optimization of dose and dosage regimen when a combination of anticancer moieties (chemotherapeutic agents and phytochemicals) are used. The Food and Drug Administration (FDA) has made changes to its regulatory policies in order to streamline the process of developing botanical drugs. This includes the introduction of a proposal for herbal IND applications to be deemed “safe to proceed” (STP) for phase 1 or 2 clinical studies. The basis for this determination is the prior human experience of these botanical drugs, either as traditional medicines (such as Traditional Chinese Medicine or Ayurveda) or through evidence of their marketing experience (such as dietary supplements). This evidence includes factors such as sales volume and reported adverse events. The implementation of a flexible regulatory approach offers the botanical industries various incentives to promote the initiation of early-phase trials for botanical products, without the requirement of additional purification of drug substances or identification of active constituents. Furthermore, this approach allows for the utilisation of existing human data as a substitute for or delay of animal toxicology studies, potentially leading to a reduction in the overall timeline for the development of botanical drugs. In order to acknowledge the intricate nature of botanical drugs and the inherent uncertainty surrounding their active constituent(s), the Food and Drug Administration (FDA) typically adheres to the Botanical Drug Development Guidance for Industry, with particular emphasis on section VI entitled “Investigational New Drug Applications for Phase 3 Clinical Studies.” This approach serves to both endorse botanicals and offer supplementary recommendations for conducting phase 3 clinical trials. After phase 3 trials, changing growth sites or adding new growing sites makes NDA filing more challenging. Prior to submitting an NDA, the FDA advises the sponsor to take into account the prospect of qualifying as many growth sites as possible. However, we hope that with the evolution of new techniques and research scientists will overcome all the challenges to treat resistant cancer in the future.

## Challenges of Herbal Nanoformulations

The prevalent cancer treatments have certain limitations like plasticity, which leads to phenotyping switching and then ultimately multi-drug-resistance (MDR) by tumors, intra-tumoral heterogenicity, tumor sites targeting of the therapy, understanding of tumor microenvironment, and insufficient drug circulation times. Besides these, these therapies are associated with cancer drug toxicity, heart problems, and low white blood cell counts.

Nanotechnology is a more advanced and immensely beneficial system than conventional therapeutics approaches. Nanotechnology involves diagnosis and therapeutics strategies in applying structures, characterization, design, devices, production, and systems at the nanometer scale. Nanocarriers have various advantages like small size (10–1000 nm), surface charge, porosity, elasticity, co-delivery of drugs and phytochemicals, enhanced solubility, prolonged systemic circulation, entrapment of lipophilic and hydrophilic drugs, easy penetration through the various biological membranes, protection of the drugs from external physiological damage by encapsulating, enhanced stability during storage by protecting the phytochemicals from oxidation and degradation, resulted into long-term effects, target-specific drug delivery thereby lowering toxicity and increasing the efficacy of the drug/phytochemicals [161]. Nano-delivery systems improve the pharmacokinetics parameters like solubility, half-life, clearance rate, and biodistribution of drug/drugs, thereby creating a therapeutic index. Small-sized NPs (less than 100 nm) are characterized by high drug loading capacity and deep penetration power into tumor tissue more effectively through enhanced vascular permeation than large-sized NPs [110]. Not only size but shape also affects as disc-shaped NPs (nanodiscs) more effectively bind to melanoma cells for more extended periods than spheroid NPs [111].

Further, breast cancer cells more effectively absorb rod-shaped NPs than spherical NPs [2]. At the same time, nanodiamonds are more effective in treating brain tumors, as they remain in the tumor longer, increasing their effectiveness [190]. The enhanced permeability and retention (EPR) effect was achieved via passive targeting. Nanocarriers are leaked through blood vessels near the tumor and retained for extended periods due to poor lymphatic drainage. If the encapsulated phytochemicals are not appropriately released sustainably or controlled, then such delivery systems are useless. For active targeting, nanocarriers are decorated with antibody fragments, peptides, growth factors, and monoclonal antibodies to recognize the overexpressed receptors or antigens by biophysical interactions. MDR is the main problem of cancer therapy that can be overcome by using nanotechnology to deliver phytochemicals.

Antibody-drug conjugates (ADCs) in the nanometer range with small-molecule cytotoxins chemically linked to an antibody that could be an excellent phytochemical carrier. Generally, biocompatible, biodegradable, and amphiphilic polymeric materials are chosen for nanocarriers that can facilitate the encapsulation of polar and nonpolar therapeutics. Some carrier systems are solid particulate types, while others are vesicle types. The particulate type solid lipid NP is widely used within the 10 to 1000 nm size range in biomedical fields. Dendrimer, again a nano- star-shaped polymeric carrier system, quickly accumulates various types of molecules as they have a central core and interior and exterior branches like a tree with multifunctional surface groups. They can also carry biomolecules (proteins and amino acids), drug molecules, and phytochemicals. Gold NPs have unique properties due to the moieties of gold and the NP system. Gold facilitates bonding with amine and thiol groups, preferred for ligand attaching in targeting, while the nano-particulate form provides a large surface area and high stability.

Depending upon the type of polymer, we can decorate it with various conjugated molecules with improved biocompatibility, less toxicity, and controlled and sustained drug release. In magnetic NPs, the magnetic field can be manipulated as controlled externally by varying the precursor concentration. As they generally agglomerate, which can be overcome by silica, polymer, or surfactant molecule’s coating that ultimately enhances dispersibility. The therapeutic efficiency of magnetic NPs in cancer cells is enhanced by lipid coating and targeted thermo-chemotherapy to folate receptors, which remain overexpressed. The carbon nanotubes (CNTs) made of rolled graphene sheets easily accommodate the therapeutic molecules as they have a narrow diameter, larger surface area, and broader length. With surface modification via oxygen-containing functional groups, ligand-targeting is possible [2].

## Future Prospects

The collaborative research conducted between traditional herbal treatments and nanotechnology, has yielded promising pharmaceutical therapies that hold potential for enhancing human health in the near future. Hence, the anticipated impact of incorporating natural products and herbal remedies alongside nanocarriers is expected to enhance the significance of current drug delivery systems in terms of their effectiveness and value.

While herbal nanoformulation presents certain challenges as discussed in previous section, it also provides an avenue for novel research opportunities [191]. The accumulation of evidence is necessary to support the notion that nanotechnology, specifically phytofabricated nano-carriers, may offer a significant solution to various challenges associated with herbal nanoformulations. Moreover, the global scale-up of these nanoformulations to enhance productivity poses a significant challenge. The tools for assessing the biological efficacy of nanoformulations are currently in the process of development in order to establish a comprehensive framework for evaluating the performance of nanoformulations. This represents a significant challenge that must be addressed in order to effectively navigate the path towards successful implementation of nanoformulations.

## Conclusion

Natural substances derived from plants have long been acknowledged as an essential source of anti-cancer medications. During the past 25 years, over 65% of new anticancer medications have come from natural sources. These naturally derived chemicals were produced in vast quantities, and their novel equivalents were created by chemical synthesis (either partial or total). The majority of these chemicals have limited bioavailability and low solubility. Additionally, the non-specificity of chemotherapy has long caused damage to the patient’s normal proliferating tissues, leaving them immunodeficient and with long-term adverse effects.

Regarding this, the emergence of nanotechnology in cancer therapies have shown promise in overcoming the drawbacks of traditional therapeutic regimens, which included barriers of instability, low aqueous solubility, high toxicity, poor absorption, low specificity, and multidrug resistance. The novel delivery systems based on nanotechnology have various benefits, including water solubility, decreased toxicity, biocompatibility, and the ability to have their surfaces modified for other purposes. Better therapeutic response and long-term survival observed in patients who received NPs have further demonstrated this. The potential for incorporating naturally occurring chemicals into nanotechnology-based combination medication formulations that target the tumor microenvironment is enormous. Studies targeted at synthesizing more tumor-targeted nanotherapeutic delivery systems with high-quality and yield cytotoxic compounds drawn from natural resources may improve the general treatment of cancer. It has been determined that combining traditional chemotherapy and plant-based drugs to display synergistic activity can result in superior treatment outcomes. It is extremely challenging for scientists to formulate nanocarriers for phytochemicals combined with chemotherapeutic medicines and optimize the dosage.

Additionally, it is challenging to coordinate their pharmacokinetics and biodistribution to keep a synergistic ratio of drugs at the tumor site. Additionally, despite phyto-nanoformulations showing promising anticancer results in preclinical research, they have not yet been developed for use in clinical settings. However, nanocarriers may exhibit some level of harm in humans based on commercial or therapeutic possibilities, even if this is unproven. Additionally, therapeutically viable phytochemical-based anticancer medicines might be made possible by effective formulation targeting methodologies, evaluation of the targeting effectiveness of NPs, and compliance with international standards for their toxicological and biocompatibility. However, scientists are confident that nanoformulations based on phytochemicals will undoubtedly find a position in the arsenal to cure cancer shortly.

## Data Availability

Not applicable.
